# Performance of models for estimating absolute risk difference in multicenter trials with binary outcome

**DOI:** 10.1186/s12874-016-0217-0

**Published:** 2016-08-30

**Authors:** Claudia Pedroza, Van Thi Thanh Truong

**Affiliations:** Center for Clinical Research and Evidence-Based Medicine, McGovern Medical School, 6431 Fannin St., MSB 2.106, Houston, 77030 TX USA

**Keywords:** Clustered data, Correlated binary data, Generalized estimating equation, Multicenter trial, Risk difference, Robust standard errors

## Abstract

**Background:**

Reporting of absolute risk difference (RD) is recommended for clinical and epidemiological prospective studies. In analyses of multicenter studies, adjustment for center is necessary when randomization is stratified by center or when there is large variation in patients outcomes across centers. While regression methods are used to estimate RD adjusted for baseline predictors and clustering, no formal evaluation of their performance has been previously conducted.

**Methods:**

We performed a simulation study to evaluate 6 regression methods fitted under a generalized estimating equation framework: binomial identity, Poisson identity, Normal identity, log binomial, log Poisson, and logistic regression model. We compared the model estimates to unadjusted estimates. We varied the true response function (identity or log), number of subjects per center, true risk difference, control outcome rate, effect of baseline predictor, and intracenter correlation. We compared the models in terms of convergence, absolute bias and coverage of 95 % confidence intervals for RD.

**Results:**

The 6 models performed very similar to each other for the majority of scenarios. However, the log binomial model did not converge for a large portion of the scenarios including a baseline predictor. In scenarios with outcome rate close to the parameter boundary, the binomial and Poisson identity models had the best performance, but differences from other models were negligible. The unadjusted method introduced little bias to the RD estimates, but its coverage was larger than the nominal value in some scenarios with an identity response. Under the log response, coverage from the unadjusted method was well below the nominal value (<80 *%*) for some scenarios.

**Conclusions:**

We recommend the use of a binomial or Poisson GEE model with identity link to estimate RD for correlated binary outcome data. If these models fail to run, then either a logistic regression, log Poisson regression, or linear regression GEE model can be used.

**Electronic supplementary material:**

The online version of this article (doi:10.1186/s12874-016-0217-0) contains supplementary material, which is available to authorized users.

## Background

Arguments have been made for reporting meaningful treatment measures, such as absolute risk difference and relative risk (RR), in clinical and epidemiological prospective studies [1–4]. For clinicians considering the likely benefits of a treatment for individual patients, the most relevant measure of treatment effect is the absolute difference in benefit or harm from two treatment options [5–7]. For binary outcomes, this corresponds to the absolute risk difference (RD) or its reciprocal, the number needed to treat [2, 5–7]. By combining the RR and the risk of the disease outcome, the number needed to treat tells us how much the treatment reduces or increases risk for an individual and hence the likelihood of benefit or harm for that individual [5, 8]. Compared to the RR, the RD is also better understood by clinicians and patients [9, 10]. For these reasons, the CONSORT statement recommends reporting of both RD and RR for all trials with binary outcomes [11].

In randomized clinical trials, estimates of RD can be obtained from analysis of a 2×2 table, but regression models are preferable when adjustment is needed for design variables (e.g., stratifying variables) or for baseline covariates that are strong predictors of outcome (to gain efficiency) [12]. In multicenter trials, adjustment for center is necessary when randomization is stratified by center or when there is large variation in patients outcomes across centers [13–15]. While the binomial model with identity link is the natural choice to obtain adjusted RD estimates, problems with convergence may limit its practical application [16, 17]. For uncorrelated binary data, an average risk difference approach was proposed as a way to calculate RD from a multiple logistic regression, controlling for covariates in observational studies [18]. This approach was shown to perform better than models with identity link regarding bias, coverage and precision [19]. Yet, comprehensive model comparisons for RD computation in the context of clustered or correlated binary data (e.g., data from a multicenter or cluster trial) have not been conducted. In the present paper, we report results of a simulation study investigating performance measures of six models for calculating RD with correlated binary data arising from a multicenter trial design. All models are fitted under a generalized estimating equations (GEE) framework, since GEE models have been shown to perform well for estimating RR and odds ratios (ORs) with correlated data.

## Methods

We consider a clinical trial study design involving *J* centers where subjects are randomized to treatment or control. We assume that outcomes from different centers are independent, but outcomes from the same center are correlated. Letting *π*(1) be the probability of the outcome with treatment and *π*(0) the probability of the outcome with the control condition, the risk difference is defined as *π*(1)−*π*(0). We can estimate this quantity with the difference of the observed proportions, $\hat {p}_{1}-\hat {p}_{0}$, or with model-based estimates to adjust for baseline covariates and clustering.

We examined six models that can be used to estimate the RD while accounting for intracenter correlation. To account for clustered data, we used GEE methods [20] to fit all models assuming an exchangeable correlation structure where subjects’ outcomes from the same center have equal correlation *ρ* with each other but are uncorrelated with outcomes from subjects in other centers. Additionally, we used robust sandwich variance estimators with small sample correction to compute standard errors (SEs) for the estimated RD [20, 21]. Robust SEs are typically used with GEE methods to account for possible misspecification of the covariance structure and distribution of the outcome (i.e., Poisson for binary outcomes).

### Models

The models evaluated used either an identity link, a log link, or a logistic link to model the probability *π*_*ij*_ of the binary outcome *y*_*ij*_.

#### Identity link models

These models assume that

1 $$\begin{array}{*{20}l}  & y_{ij} \sim \mathrm{F}(\pi_{ij})  \\ & \pi_{ij} = \alpha+ \beta\,{x_{ij}}+ \gamma\,{z_{ij}} \end{array} $$

where *y*_*ij*_ is the binary outcome for subject *i* (*i*=1,2,…,*n*_*j*_) in center *j* (*j*=1,2,…,*J*) with probability *π*_*ij*_, and F (·) is either a binomial, Poisson, or Normal distribution; *x*_*ij*_ is a binary indicator for treatment and *z*_*ij*_ is a binary baseline covariate. The regression coefficient for the treatment variable, *β*, is the estimated RD between the treatment and control groups adjusting for the baseline covariate and accounting for intracenter correlation. The main disadvantage of these models is that the estimate of *π*_*ij*_ may be outside of the [0, 1] range (and may not be useful for estimating individual risks), and the binomial model may have convergence problems. However, a direct estimate of the RD is obtained from *β*.

#### Log link models

Here we assume that

2 $$\begin{array}{*{20}l}  & y_{ij} \sim \mathrm{F}(\pi_{ij})  \\ &\text{log} (\pi_{ij}) = \alpha + \beta\,{x_{ij}}+ \gamma\,{z_{ij}}, \end{array} $$

where the *β* is the log RR of the disease comparing treatment to control. For this link, we used a binomial or Poisson distribution for *y*_*ij*_. The log Poisson model is typically used to estimate RRs since it tends to be more stable than the log binomial model and gives consistent estimates [16]. However, similar to the identity link models the estimate of *π*_*ij*_ resulting from the log link models may be larger than 1.

To estimate the average RD, we use Eq. 2 to estimate subject-specific probabilities of the outcome under the treatment and control conditions. We calculate the probability of the outcome if the subject is treated as $\hat \pi _{ij}(1)=\text {exp} (\hat \alpha + \hat \beta \,{x_{ij}}+ \hat \gamma \, {z_{ij}})$, and $\hat \pi _{ij}(0)=\text {exp} (\hat \alpha + \hat \gamma \, {z_{ij}})$ if the subject does not receive treatment. We then estimate the average RD for the whole study population as

3 $$ \widehat{\text{RD}} = \frac{1}{n}\sum_{ij}(\hat\pi_{ij}(1)-\hat\pi_{ij}(0))  $$

where *n* is the total sample size.

#### Logit link model

Here we use the logistic regression model assuming that

4 $$\begin{array}{*{20}l} & y_{ij} \sim \text{Bernoulli}(\pi_{ij})  \\ & \text{logit}(\pi_{ij}) = \alpha + \beta\,{x_{ij}}+ \gamma\,{z_{ij}}. \end{array} $$

The coefficient *β* is the log odds ratio. Similar to the log link models, we can use the logistic model 4 to estimate the probability of the outcome for each subject under treatment or control condition. For a given subject, the probability of the outcome with treatment is

5 $$ \hat\pi_{ij}(1)= \frac{\text{exp}(\hat \alpha + \hat \beta\,{x_{ij}}+ \hat\gamma\, {z_{ij}})}{1+\text{exp} (\hat \alpha + \hat \beta\,{x_{ij}}+ \hat\gamma\, {z_{ij}})}  $$

and

6 $$ \hat\pi_{ij}(0)=\frac{\text{exp}(\hat\alpha + \hat{\gamma}\,{z_{ij}})}{1+\text{exp}(\hat\alpha + \hat{\gamma}\,{z_{ij}})} $$

with no treatment. The average RD is again estimated with Eq. 3.

#### Standard errors of the RD

For models with identity link, the SE for the RD was obtained from the fitted model. For models with a log or logit link, standard errors of the RD were calculated using the delta method. A sampling correction factor of *J*/(*J*−*p*−1), where *J* is the number of centers and *p* is the number of variables in the model, was applied to robust SEs from all regression models to account for the small number of centers [21]. We used this corrected SE to compute Wald-type 95 % confidence intervals (CIs) for the RD: ${\widehat {\text {RD}}} \pm 1.96\times {\text {SE}_{\text {corrected}}(\widehat {\text {RD}})}.$

## Simulation study

We assumed a multicenter randomized trial study design with 18 centers to assess performance of the models with a small number of centers (i.e., <50), which is common in perinatal trials [22–24]. Randomization is stratified by center using blocks of size four with approximately equal number of subjects per center. We considered two sets of different simulation scenarios. The first set assumes a true identity link response function as in (1), and the second set assumes a true log link function shown in (2). We include the log function to check the robustness of the models since we cannot know whether a real data set comes from an additive or multiplicative model. For both sets of scenarios, we varied the number of subjects per center (*n*_*j*_), the outcome rate in the control group (*π*_*c*_), intracenter correlation coefficient (ICC), true RD, and the effect of the baseline covariate on the outcome.

### Data generation with true identity link function

We simulated 1000 trial datasets for each combination of the parameters using the model with identity link function:

7 $$\begin{array}{*{20}l} & y_{ij} \sim\text{Bernoulli}(\pi_{ij})  \\ & \pi_{ij} = \alpha+ \beta\,{x_{ij}}+ \gamma\,{z_{ij}} + \nu_{j}. \end{array} $$

The treatment indicator *x*_*ij*_ was generated by randomization stratified by center, covariate *z*_*ij*_ from Bernoulli (0.3), and random center effect *ν*_*j*_ from Normal (0,*σ*^2^) to induce the center correlation. The number of subjects per center *n*_*j*_ was 10, 50, or 100 (total sample sizes of 180, 900, or 1800) corresponding to small, medium, and large sample sizes. Outcome rate in the control group, *α*=*π*_*c*_, was 0.10, 0.25, or 0.50. Values of *β*=0,0.05,0.10, and 0.15 corresponding to true RDs ranging from no effect to a large treatment effect were considered. The covariate effect *γ* was 0.5*α* or 0 corresponding to a 50 % increased risk in the presence of the covariate (an effect strong enough such that it would be included in the design or analysis) or no effect (i.e., no baseline covariate). The ICC *ρ* was set to 0.01, 0.05, or 0.10 which are values typically found in clinical cluster trials [25, 26]. We calculated the center variance as $\sigma ^{2}=\rho \,{\bar {\pi }}(1-\bar {\pi })$, where $\bar {\pi }=\alpha +0.5\beta +0.3\gamma $ is the average probability of the outcome among the entire study population [27]. The values of *σ*^2^ ranged from 0.001 to 0.025. Whenever the simulated *π*_*ij*_ was outside the [0,1] interval, a new value of the random center effect *ν*_*j*_ was sampled until 0<*π*_*ij*_<1.

### Data generation with true log link function

Since our main interest is in the estimation of the average RD, we assume the same true RD (0, 0.05, 0.10, 0.15) and control outcome rate *π*_*c*_ (0.10, 0.25, 0.50) values as for the identity response simulation set. We generated 1000 datasets from the log link function model:

8 $$\begin{array}{*{20}l} & y_{ij} \sim\text{Bernoulli}(\pi_{ij})  \\ & \log(\pi_{ij}) = \alpha+ \beta\,{x_{ij}}+ \gamma\,{z_{ij}}+ \nu_{j}. \end{array} $$

The *x*_*ij*_’s were obtained from the randomization; *z*_*ij*_’s, and *ν*_*j*_’s were generated from a Bernoulli (0.3) and Normal (0,*σ*^2^), respectively. ICC values of 0.01, 0.05, or 0.10 were again used for *ρ*, and *n*_*j*_=10,50,100. For the log link function, we calculated the center variance based on a variance transformation approximation [27, 28] with $\sigma ^{2}=\rho \,(1-\bar {\pi })/{\bar {\pi }}$, where $\bar {\pi }=\text {exp}(\alpha +0.5\beta +0.3\gamma)$. Values of *σ*^2^ ranged from 0.006 to 0.90. To correspond with the values used in the identity response simulation set, the parameter values in model (8) were *α*= log(0.1), log(0.25), log(0.5); *γ*= log(1.5), 0; and *β*=log(1+RD/exp(*α*+0.3*γ*)). We again restricted the simulated *π*_*ij*_ to the [0,1] interval by generating a new value of *ν*_*j*_ whenever the *π*_*ij*_ fell outside this interval.

Using a fully factorial study design, we investigated a total of 432 simulation scenarios (2 response functions × 4 true RDs × 3 control outcome rates × 3 ICCs × 3 sample sizes × 2 covariate effects). All the simulations and computations were performed in R 3.1.1 [29]. All models were implemented using the geese function in the R package geepack [30–32]. Unadjusted RD estimates (and CIs) were obtained using the epi.2by2 function from the epiR package [33]. We provide sample code in the Additional file 1 for calculating the RD and SEs from all GEE models for scenarios with covariate adjustment.

### Performance measures

Under each simulation scenario, we analyzed each of the 1000 data sets with the six GEE models and the unadjusted method (to evaluate the impact of ignoring the intracenter correlation): 1) binomial identity; 2) Poisson identity; 3) Normal identity; 4) log binomial; 5) log Poisson; 6) logistic; 7) unadjusted 2×2 table analysis. We computed point estimates, SEs, and 95 % CIs for the RD. We evaluated and compared the different models based on convergence rate (model runs and converges), absolute bias, and coverage of the 95 % CI. Absolute bias was calculated as the average difference between the estimated RD and the true RD. The coverage of the 95 % CI is the proportion of simulations resulting in 95 % CIs for the RD that include the true RD. When computing bias and coverage for a model, we only included the datasets for which that specific model converged.

## Results

**Fig. 1:**
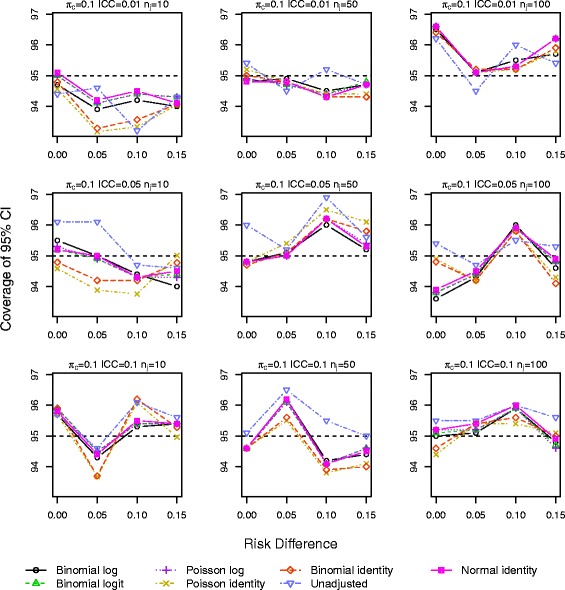
Coverage of 95 % CIs for scenarios with true identity link function including a baseline covariate for control outcome rate *π*_*c*_=0.10

**Fig. 2:**
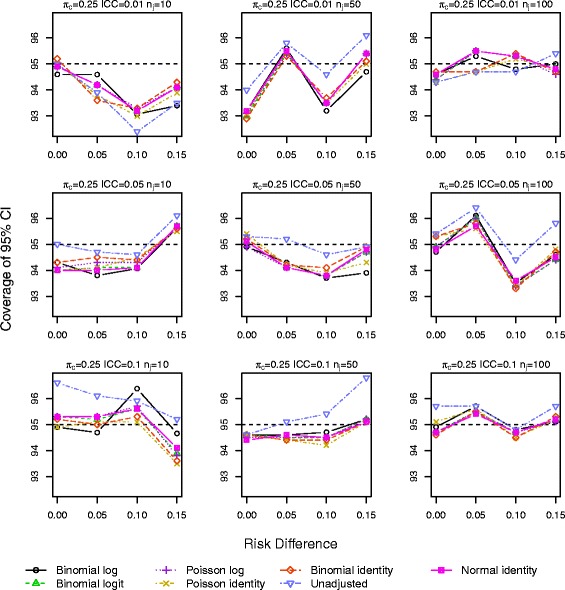
Coverage of 95 % CIs for scenarios with true identity link function including a baseline covariate for control outcome rate *π*_*c*_=0.25

**Fig. 3:**
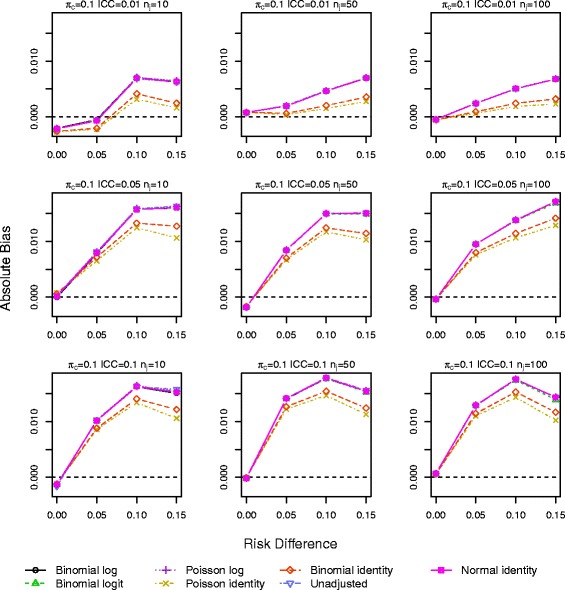
Absolute bias for scenarios with true log link function including a baseline covariate for control outcome rate *π*_*c*_=0.10

**Fig. 4:**
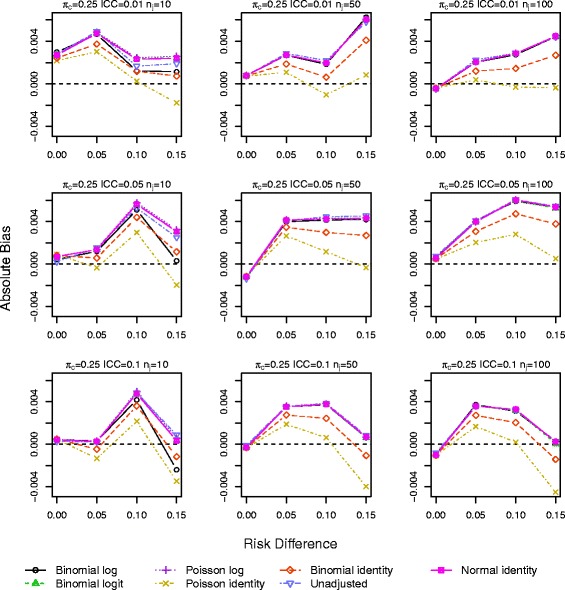
Absolute bias for scenarios with true log link function including a baseline covariate for control outcome rate *π*_*c*_=0.25

**Fig. 5:**
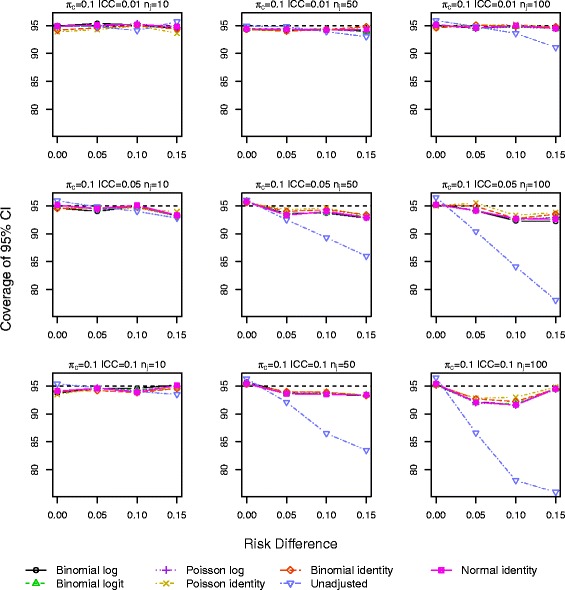
Coverage of 95 % CIs for scenarios with true log link function including a baseline covariate for control outcome rate *π*_*c*_=0.10

**Fig. 6:**
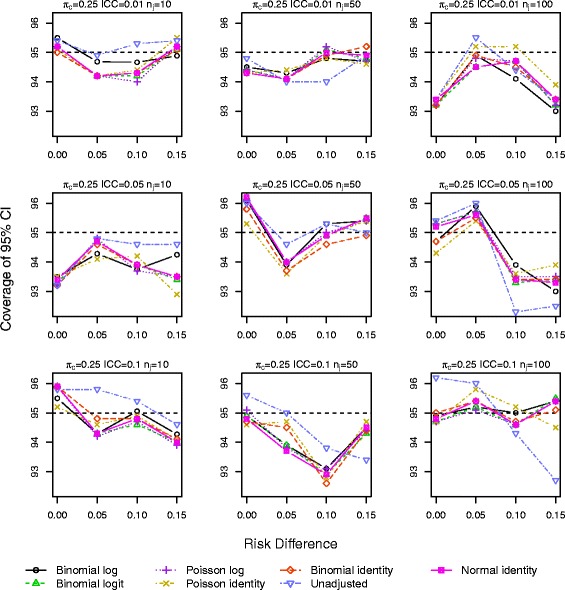
Coverage of 95 % CIs for scenarios with true log link function including a baseline covariate for control outcome rate *π*_*c*_=0.25

Results for *π*_*c*_=0.10 and 0.25 with a baseline covariate are shown in Figs. 1, 2, 3, 4, 5 and 6. Graphs of the results for all other simulation scenarios are included in the Additional file 1.

### True identity link function

#### Scenarios without a baseline covariate

All models ran without errors. Absolute bias from all models is virtually the same with magnitude of ± 0.004 for *n*_*j*_=10 and even smaller for the larger sample sizes. Coverage of the 95 % CIs was within the 95 % nominal level (93.6–96.4 %) for the majority of scenarios and models. However for some scenarios with smaller values of ICC, small sample size, or large true RD, the coverage of all the GEE models was below 93.6 % (minimum coverage was 92 %). For the same scenarios the coverage of the unadjusted method tended to also be low (lowest coverage was also 92 %). Conversely, for some scenarios with the largest ICC of 0.10, the coverage of the unadjusted RD estimates was larger than the nominal value indicating conservative CIs (maximum coverage was 97 %).

#### Scenarios with a baseline covariate

The log binomial model failed to converge for a large portion of the datasets for scenarios with *π*_*c*_=0.50 and hence we do not report results from this model for this control rate. For *π*_*c*_=0.10,0.25, the log binomial, binomial identity, and Poisson identity models did not converge for small proportions of the datasets (<3 *%*,<3 *%*,≤3 *%*). The point estimates of RD from all the GEE models were very similar with very little bias, especially as the sample size increases (Additional file 1: Figures S.7–S.9). The coverage of the 95 % CIs is very similar for all the GEE models, and all are close to the nominal level (Figs. 1 and 2). Again for a few scenarios with the largest ICC (Fig. 2), the coverage of the CI for the unadjusted RD estimate is larger than the nominal value.

### True log link function

#### Scenarios without a baseline covariate

All models ran without errors, and point estimates of RD from all models were the same. Here, the bias increases as the true RD increases for *π*_*c*_=0.10,0.25 with maximum bias of 0.03 and 0.01, respectively (Additional file 1: Figures S.11–S.13). Coverage probability from all GEE models is very similar and close to the 95 % nominal value, although the coverage is below 90 % for a few scenarios with *π*_*c*_=0.10 and ICC ≥0.05. The unadjusted method has poor coverage (62–90 %) for ICC >0.01 and *n*_*j*_>10 when *π*_*c*_=0.10,0.25 (see Additional file 1: Figures S.14–S.15). These scenarios correspond to larger values of center variance (*σ*^2^>0.07) where the unadjusted model vastly underestimates the SE of the RD.

#### Scenarios with a baseline covariate

The log binomial model again failed to converge for a large portion of datasets for *π*_*c*_=0.50, and we again exclude it from the results. The identity Poisson and binomial models also failed to converge for scenarios with the smallest sample size (<3 and <4 *%*, respectively). All other models ran without errors for all datasets. Bias from all models is very similar and increases as RD and ICC increase (Figs. 3 and 4). The smallest bias resulted from the Poisson identity model for scenarios with *π*_*c*_=0.10,0.25 and from the binomial identity model when *π*_*c*_=0.5 and ICC≥0.05, although differences between the models are small.

Coverage varied depending on the true *π*_*c*_. For scenarios with *π*_*c*_=0.10, with the exception of the unadjusted method all other models resulted in similar coverage very close to the nominal value. The unadjusted method had very poor coverage (≤90 *%*) when ICC >0.01 and *n*_*j*_≥50 (Fig. 5), which again correspond to scenarios with larger values of *σ*^2^(>0.24). For scenarios with *π*_*c*_=0.25, all models had good coverage although the unadjusted method had lower coverage for ICC >0.01,*n*_*j*_=100 and RD=0.15 (*σ*^2^>0.10; Fig. 6). For *π*_*c*_=0.50, all models had low coverage (86–93 %) for RD = 0.15, ICC >0.05, and *n*_*j*_≥50(*σ*^2^>0.06; Additional file 1: Figure S.18). For these scenarios, the probability of the outcome in the treatment and covariate group is close to the parameter boundary of one. In these instances, the binomial identity model had the coverage closest to nominal.

## Applications

We present two examples to illustrate the similar performance of the GEE models in randomized studies with slightly different designs than those studied in the simulations. The first example is a cluster randomized trial, and the second is a multicenter trial with small number of centers.

### Cluster trial example

The ASSIST trial assessed the effectiveness of three different interventions for improving the secondary preventive care of patients with coronary heart disease delivered at the level of general practice [34]. This study was a cluster trial where 21 general practices were randomized to the three interventions. The control group received audit of notes with summary feedback to primary health care team. The intervention groups received assistance with setting up a disease register and systematic recall of patients to either general practitioner or a nurse led clinic. The primary outcome was adequate assessment of 3 risk factors (blood pressure, cholesterol, and smoking status) at 18 months follow-up. We analyzed the data presented by Thompson et al. [27] using all 6 GEE models and the unadjusted method. We grouped the 2 intervention groups together and compared them to control. The number of patients in each practice ranged from 25 to 222 and the observed proportion of the outcome ranged from 0.38−0.73 in control practices and 0.54−0.95 in intervention ones. The point estimates and 95 % CI of the RD from all the regression models are identical (0.315, 95 % CI: 0.194, 0.436), and they are very similar to the Bayesian estimates reported by Thompson et al. (0.301, 95 % CI: 0.175, 0.435). The unadjusted method gives a lower estimate of the RD with a narrower CI (0.285, 95 % CI: 0.238, 0.331). However, the conclusions drawn from any of these estimates would most likely be the same.

### Multicenter trial with small number of centers

To compare model estimates in a study with a small number of centers, we also analyzed the data presented by Beitler and Landis [35] arising from an 8 center randomized trial. The study evaluated the efficacy of an active drug compared to control in treating an infection. The rate of success in the active drug group varied from 9–80 % in the 8 centers whereas the control group had rate of success from 0–86 % (Table 1). We fitted the 6 GEE models as well as the unadjusted method to data from 273 subjects from the 8 study centers. Table 2 shows the estimated RD from the different methods. All 6 GEE models give almost identical RD estimates (0.125–0.127) and SEs as well as estimates of ICC. The 95 % CIs from the GEE models do not cross 0 and would be considered statistically significant. In comparison, the unadjusted RD estimate is lower and not significant (0.094, 95 % CI: –0.20, 0.21) although the SE is the same as from the GEE models. This difference in RD estimates from the GEE and unadjusted model is in part due to the large variability in the response rate across the 8 centers (and hence large ICC of 0.22), which is accounted for in the GEE models. As pointed out by a reviewer, this difference can be largely accounted for by the imbalance in the number of patients in each arm across the centers. When we exclude the center with the largest baseline difference (center 2 with 20 active vs 32 control patients), the estimated RDs are very close (0.133 from GEE models vs 0.129 from unadjusted model).

**Table 1:** Data from a multicenter trial comparing the efficacy of an active drug and control for curing an infection [35]

	Response	
Center	Active No./Total (%)	Control No./Total (%)
1	11/36 (31)	10/37 (27)
2	16/20 (80)	22/32 (69)
3	14/19 (74)	7/19 (37)
4	2/16 (13)	1/17 (6)
5	6/17 (35)	0/12 (0)
6	1/11 (9)	0/10 (0)
7	1/5 (20)	1/9 (11)
8	4/6 (67)	6/7 (86)

**Table 2:** Estimates of risk difference (RD) for a multicenter trial comparing active drug and control group presented in Table 1

Model	RD	SE	95 % Confidence interval	ICC
Binomial identity	0.126	0.059	0.011, 0.241	0.218
Poisson identity	0.125	0.058	0.012, 0.238	0.219
Normal identity	0.127	0.060	0.011, 0.244	0.217
Binomial log	0.126	0.059	0.011, 0.241	0.218
Poisson log	0.125	0.058	0.012, 0.238	0.219
Binomial logit	0.126	0.059	0.011, 0.241	0.218
Unadjusted	0.094	0.058	–0.020, 0.209	–

## Discussion

In clinical and epidemiological studies with binary outcomes, it is preferable to report the RD or the number needed to treat since these effect measures are more easily understood compared to relative risks or odds ratios [5–7]. For patients and clinicians alike, it is much easier to weigh the treatment benefits and risks using the absolute RD than using the RR [36]. For multicenter studies, it is important to adjust for possible center correlation when computing treatment effects, particularly when center variability is large or when randomization is stratified by center since unadjusted SEs will be too large and lead to diminished power and possibly erroneous conclusions [13–15]. While regression methods have been proposed to estimate RDs in cluster trials [27, 37], no studies have been previously conducted to evaluate the performance of the most widely used methods, particularly in multicenter studies with a small number of centers (<50).

In this paper, we evaluated the performance of six different GEE models to estimate both unadjusted and covariate-adjusted RD while accounting for within-center correlation. We compared all models in terms of absolute bias and coverage under various simulation scenarios. We also assessed the convergence rate for all the methods since non-convergence is a known issue for the binomial with log and identity links as well as for the Poisson with identity link [16, 38]. We assessed the robustness of the methods to model misspecification by assuming a true log response function as well as an identity response. For scenarios without a covariate under both response functions, all regression models converged and performed equally well regarding absolute bias and coverage of the 95 % CIs. The unadjusted method introduced little bias to the RD estimates, but its coverage was larger than the nominal value in some scenarios with an identity response. Under the log response, coverage from the unadjusted method was well below the nominal value (<80 *%*) for scenarios with larger values of center variation where the unadjusted model underestimates the SE of the estimated risk difference. Even with the small sample correction factor, the CIs from the GEE models also had coverage lower than 95 % in some scenarios. Although the coverage obtained here is similar to that reported for estimating RRs [16, 39, 40], other variance adjustment methods to correct for the small number of clusters [21] or bootstrap estimates [41] may perform better. Alternatively, model-based SEs may be another option for GEE models [13].

For scenarios where a baseline predictor was included, the log binomial model did not converge for a large portion of the scenarios evaluated. This problem has been widely experienced and noted in both RCTs and observational studies with either independent or clustered data [16, 39, 40]. The binomial and Poisson with identity models also failed to converge for some data sets (<4 *%*) with small sample size.

While the logistic model performed very similar to the other GEE models, it might provide some challenges to analysts since the estimation of the RD is not straightforward as it is for models with an identity link. Furthermore, SEs are not directly obtained from the output and require extra steps to compute. We provide sample code in the Additional file 1 using the delta method. The linear regression model is not usually considered for binary data. However, for estimation of RD it appears to be a possible solution when paired with robust SEs. It has been previously evaluated for uncorrelated data with similar performance as the results shown here [38]. Given its ease of implementation in existing statistical software, consideration might be given to this method if a binomial or Poisson with identity link model fails to converge.

We focused on comparing GEE methods since they are often used for correlated or clustered data and have been shown to perform well for estimating RRs and ORs [13, 26, 37, 40, 42]. However, generalized linear mixed models or random effects models may also be used to analyze correlated data. Estimates derived from random effects models are interpreted as center-specific as opposed to population-averaged (or marginal) interpretation of GEE estimates. We note that under an identity link, the RD point estimates from GEE and random effects models are the same [43, 44]. However, it would be important to investigate differences in the SEs and evaluate the performance of random effect models with logit or log links for estimating RD for correlated binary outcomes.

Our results from the binomial with identity link GEE model are similar to those obtained in a study by Ukoumunne et al. [37] using the same model to analyze data from cluster trials. Given the good performance of GEE models for estimating RRs and ORs in CRTs [26, 37, 42, 45], we would expect the GEE models with identity link to perform well when estimating RD for cluster trial data, but it is unclear how the other links would perform in this setting.

Our simulation study was limited to one number of centers and an equal number of subjects within each center. However, for logit and log link GEE models others have noted similar performance when the number of centers and distribution of subjects within centers were varied [13, 39, 40, 45]. These models have also been shown to perform well with as few as 5 centers [13]. We did not consider fixed effects models since for binary outcomes these methods require small number of centers (i.e., 2–3) otherwise the treatment effect estimate can be biased and the type I error rate inflated [13]. Treating center as a fixed effect can also lead to exclusion of patients in centers that do not have adequate number of patients or events [46].

Our study evaluated a randomized trial design. However, we would expect center variability or within-center correlation to be a larger issue for observational studies where it is not unusual for centers to differ in the interventions used as well as their patient populations [13, 47]. While it is unusual for more than one baseline covariate to be included in the primary analysis of an RCT, adjustment for more than one covariate or alternative methods such as propensity scores may be needed in an observational study setting, and the methods evaluated here may perform differently in these scenarios.

We also did not consider the presence of an interaction between the intervention and a covariate or intervention and center. These situations are important, particularly if we are trying to evaluate heterogeneity of treatment effect. We recommend that these issues be evaluated in future studies.

## Conclusion

In conclusion, we recommend adjusting for center in multicenter studies. In an RCT setting with small number of centers, GEE regression models perform well for the estimation of RD, even under a misspecified model. Our results support the use of a binomial or Poisson GEE model with identity link and robust variance estimates. In cases where these models fail to run either a logistic regression, log Poisson regression, or linear regression GEE model with exchangeable correlation and robust standard errors (with small sample size correction if number of centers is <50) can be used to estimate the risk difference with correlated binary data. When preparing statistical analysis plans, we would recommend to state an alternative method of analyses in case of non-convergence of the primary method.

## Additional file

Additional file 1 Additional results and sample R code. (PDF 275 kb)

## Abbreviations

CI: Confidence interval
GEE: Generalized estimating equation
ICC: Intracenter correlation coefficient
OR: Odds ratio
RCT: Randomized controlled trial
RD: Risk difference
RR: relative risk
SE: Standard error
