# Supplementary material for: Performance of models for estimating absolute risk difference in multicenter trials with binary outcome
Source: BMC Med Res Methodol. 2016 Aug 30;16(1):113. doi: 10.1186/s12874-016-0217-0 (PMC5006411; doi:10.1186/s12874-016-0217-0)
Supplement: Additional file 1 — Additional results and sample R code. (PDF 275 kb) [file 12874_2016_217_MOESM1_ESM.pdf]

# Additional file 1 for *Performance of models for estimating absolute risk difference in multicenter trials with binary outcome*

## 1 Simulation Results

Scenarios with true identity link function without a baseline covariate: Figures S.1–S.6.
Scenarios with true identity link function including a baseline covariate: Figures S.7–S.10.
Scenarios with true log link function without a baseline covariate: Figures S.11–S.16.
Scenarios with true log link function including a baseline covariate: Figures S.17–S.18.

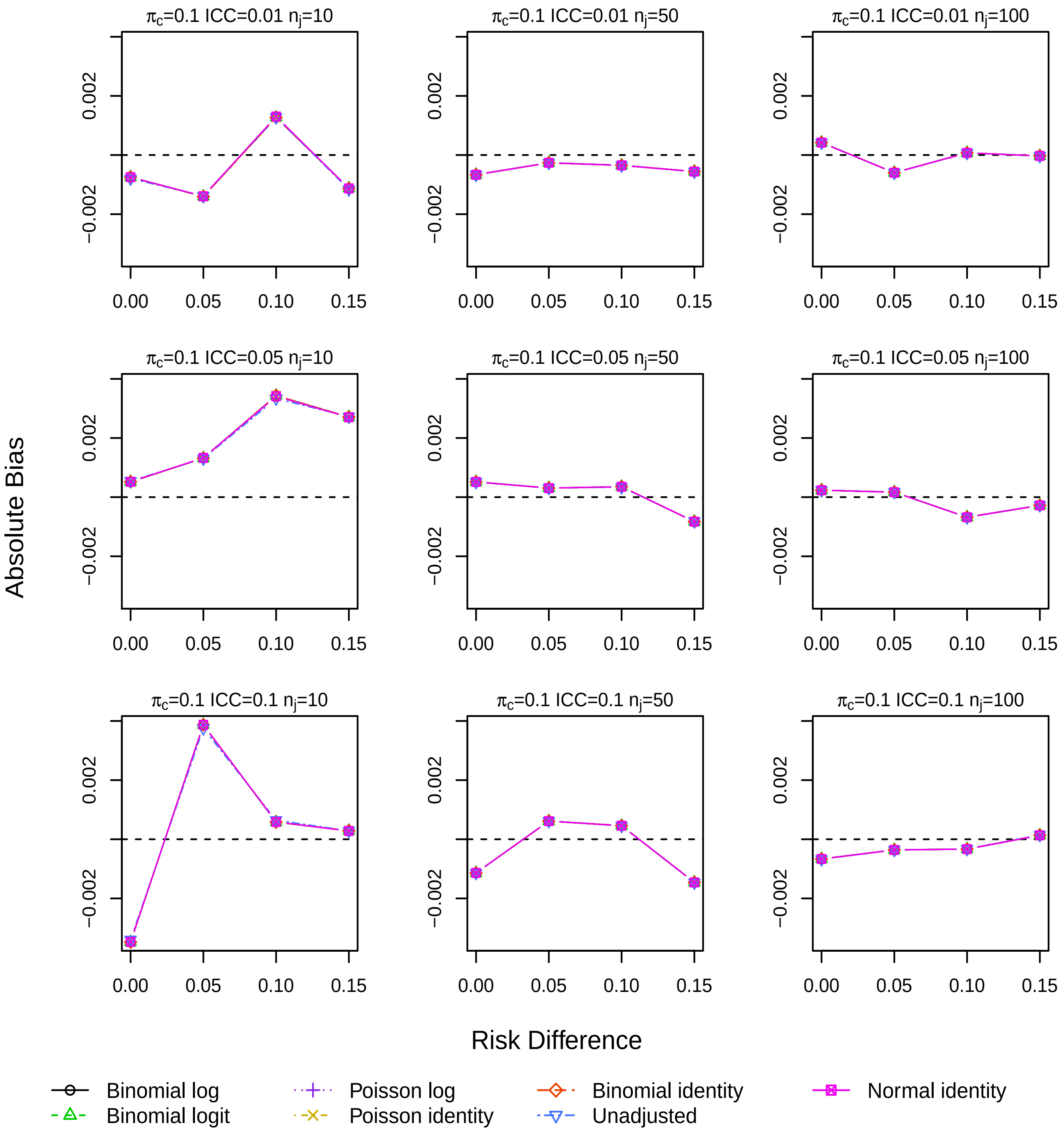


Figure S.1. Absolute bias for scenarios with true identity link function without a baseline covariate for $\pi_c = 0.10$.

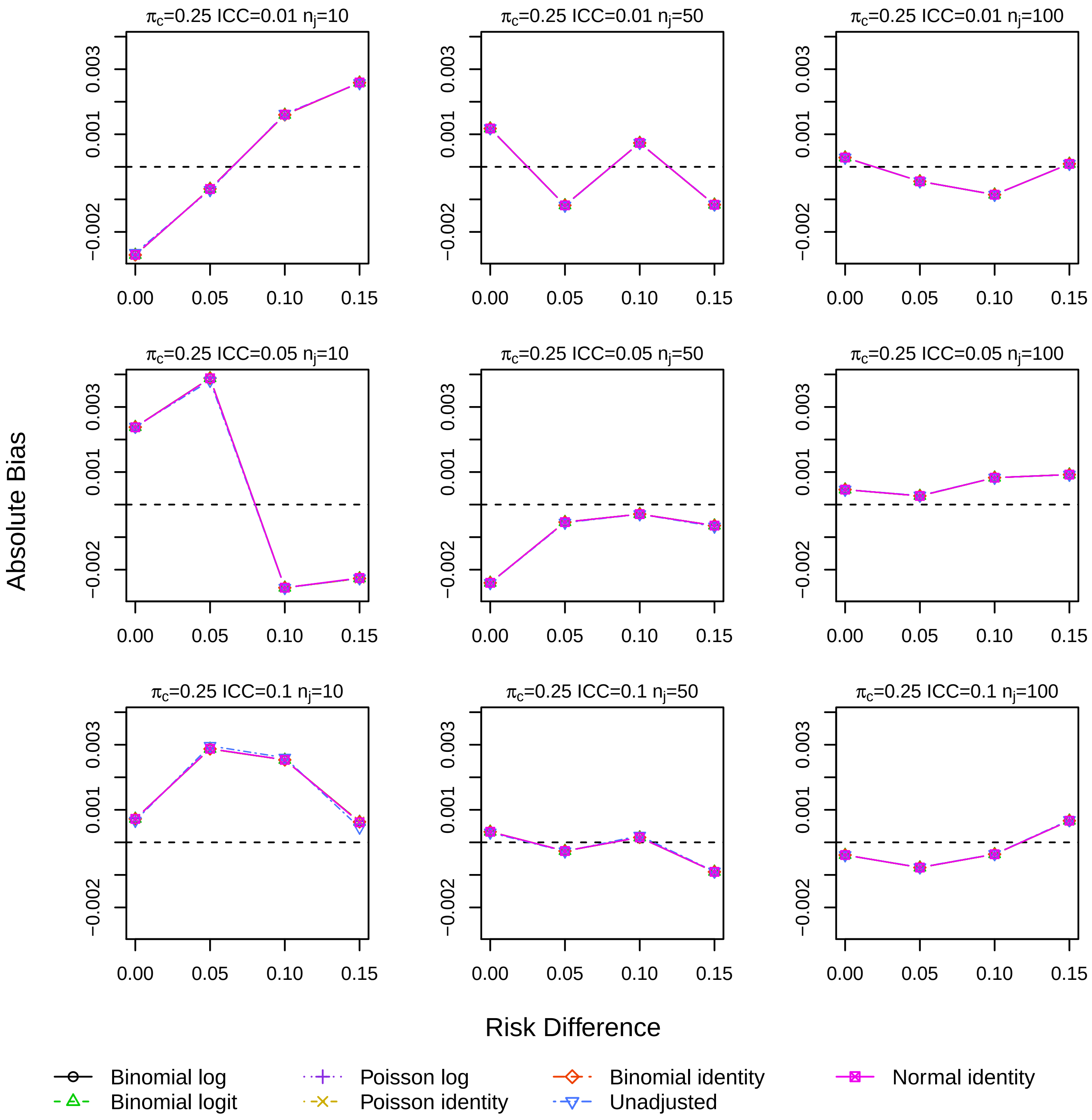


Figure S.2. Absolute bias for scenarios with true identity link function without a baseline covariate for $\pi_c = 0.25$.

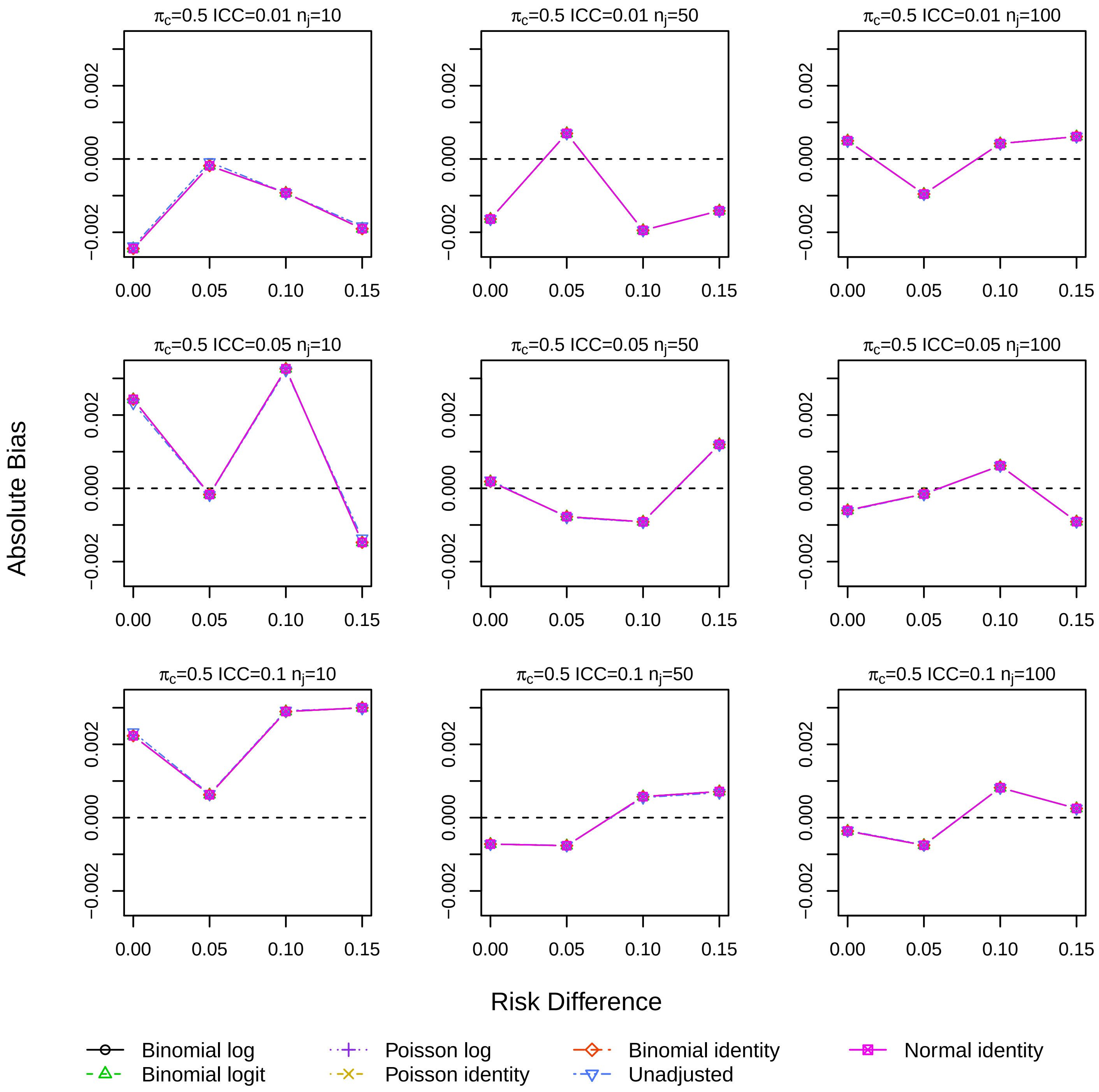


Figure S.3. Absolute bias for scenarios with true identity link function without a baseline covariate for $\pi_c = 0.50$.

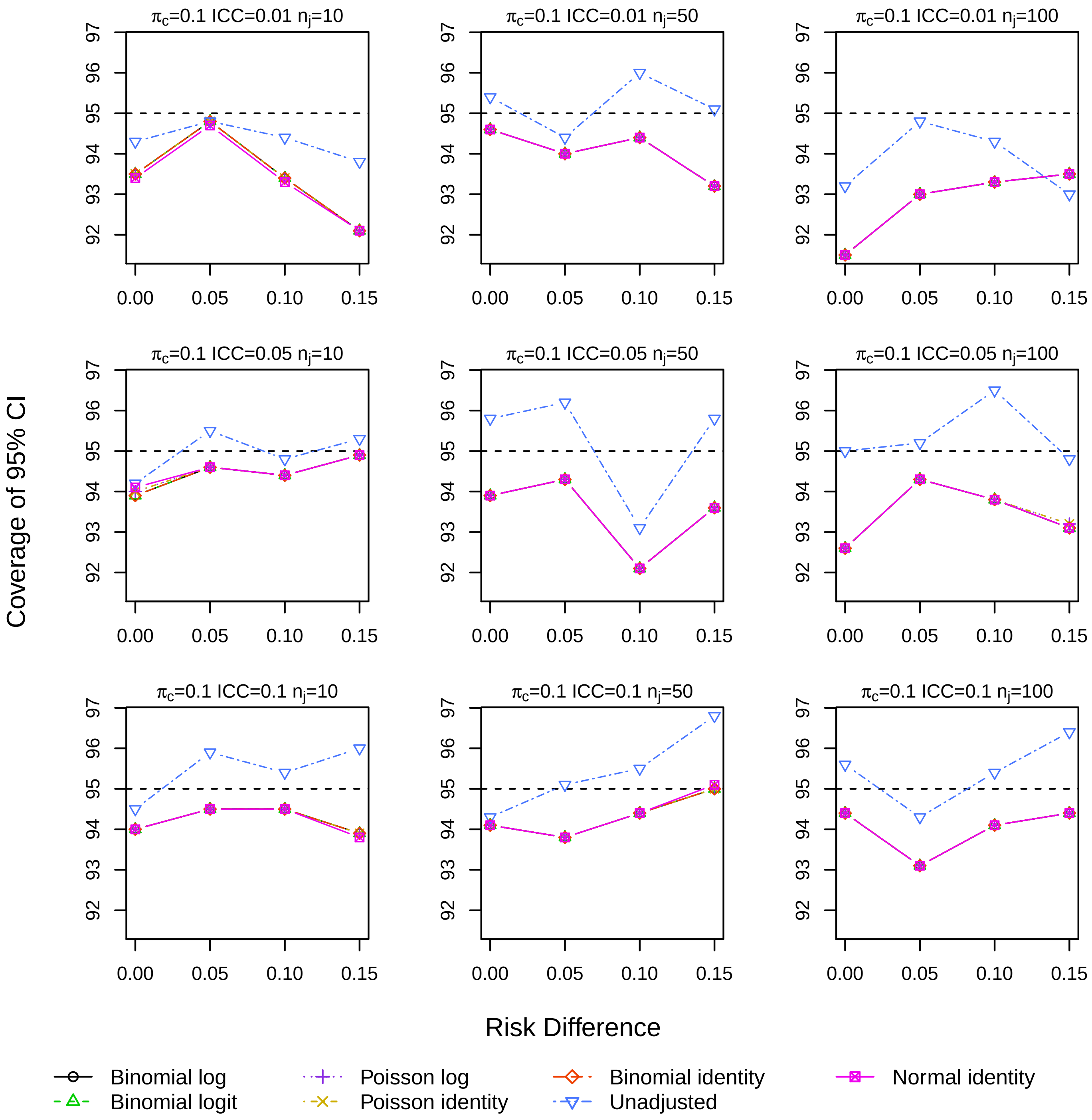


Figure S.4. Coverage of 95% CIs for scenarios with true identity link function without a baseline covariate for $\pi_c = 0.10$.

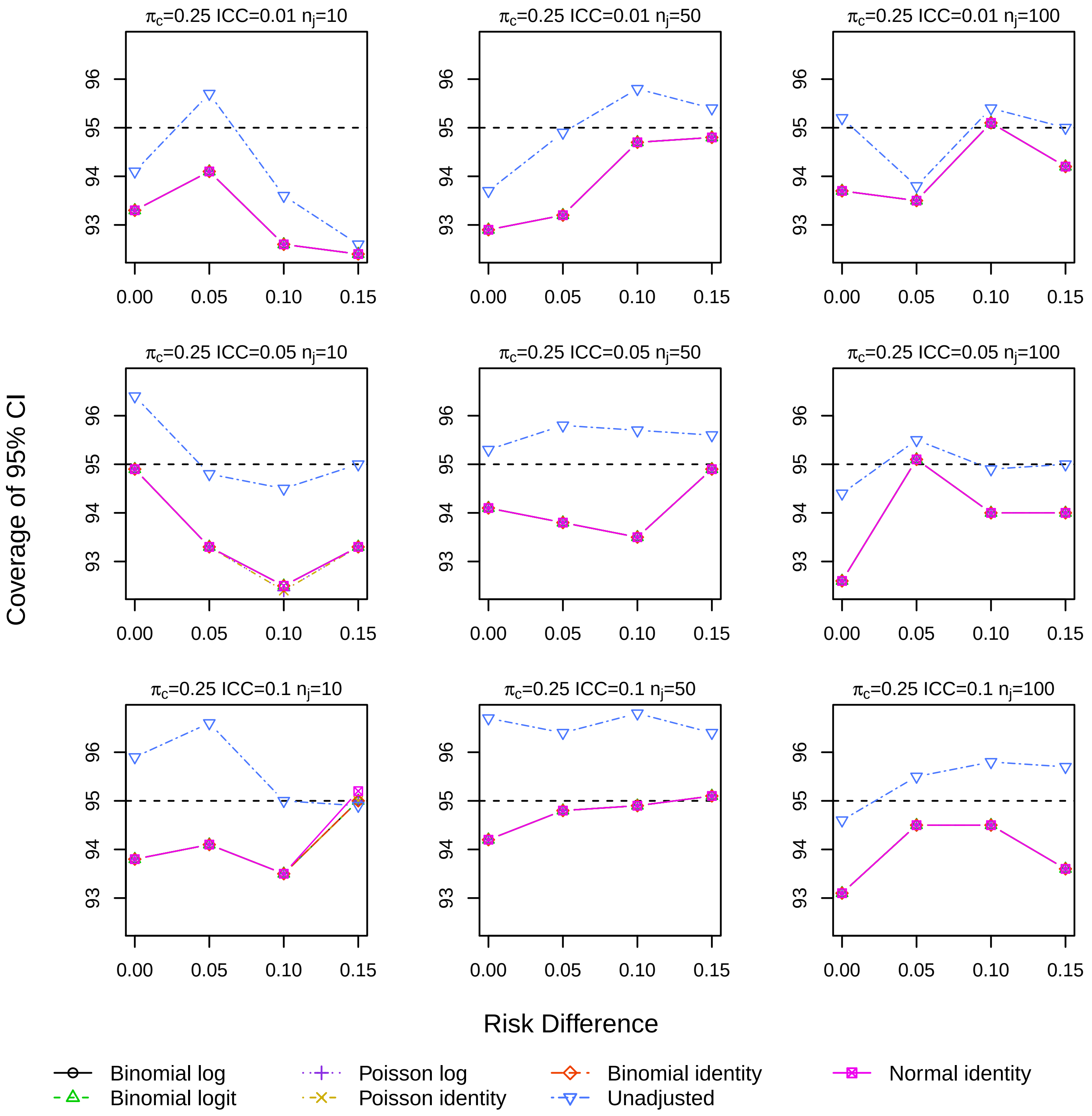


Figure S.5. Coverage of 95% CIs for scenarios with true identity link function without a baseline covariate for $\pi_c = 0.25$.

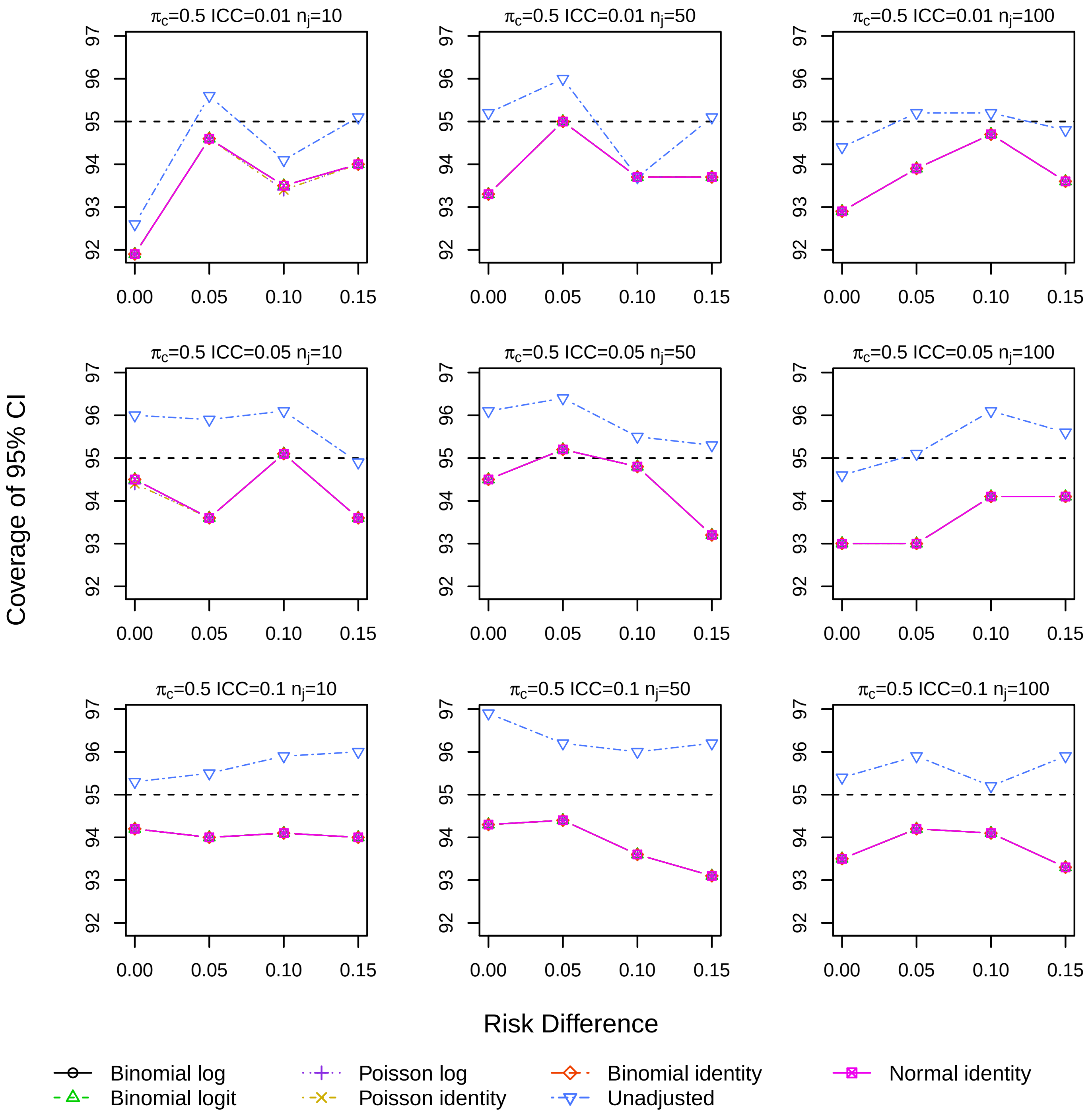


Figure S.6. Coverage of 95% CIs for scenarios with true identity link function without a baseline covariate for $\pi_c = 0.50$.

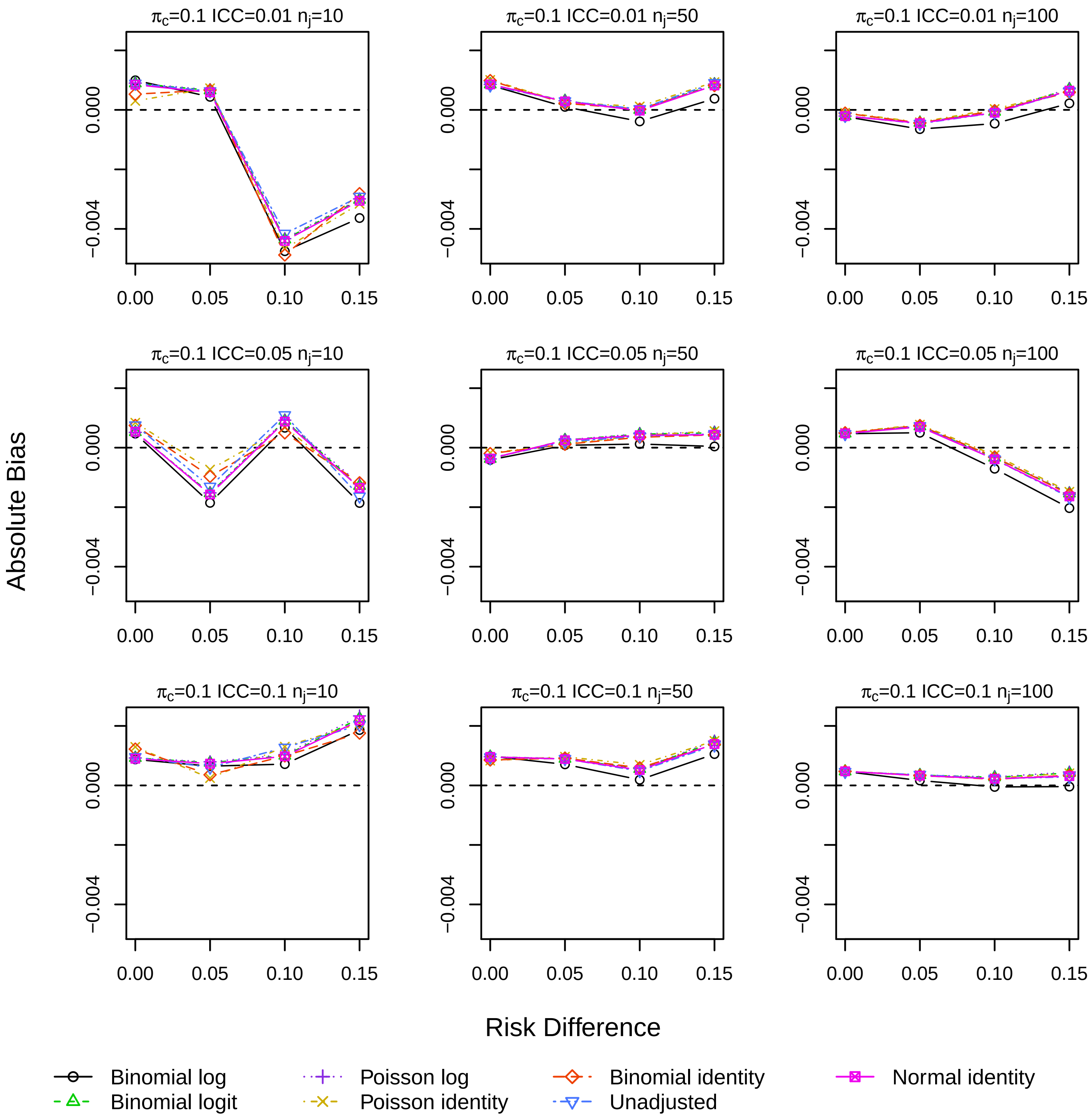


Figure S.7. Absolute bias for scenarios with true identity link function including a baseline covariate for $\pi_c = 0.10$.

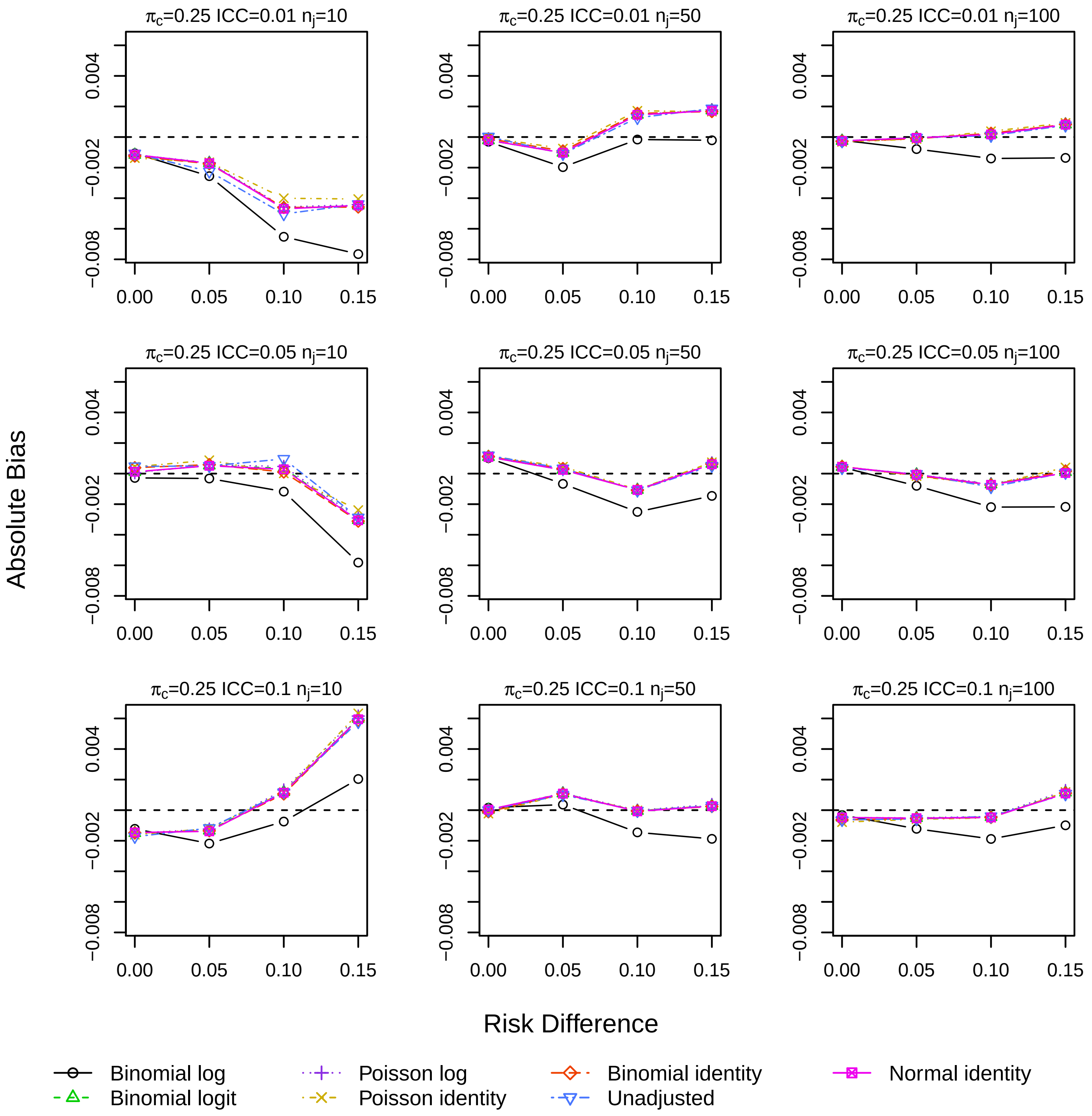


Figure S.8. Absolute bias for scenarios with true identity link function including a baseline covariate for $\pi_c = 0.25$.

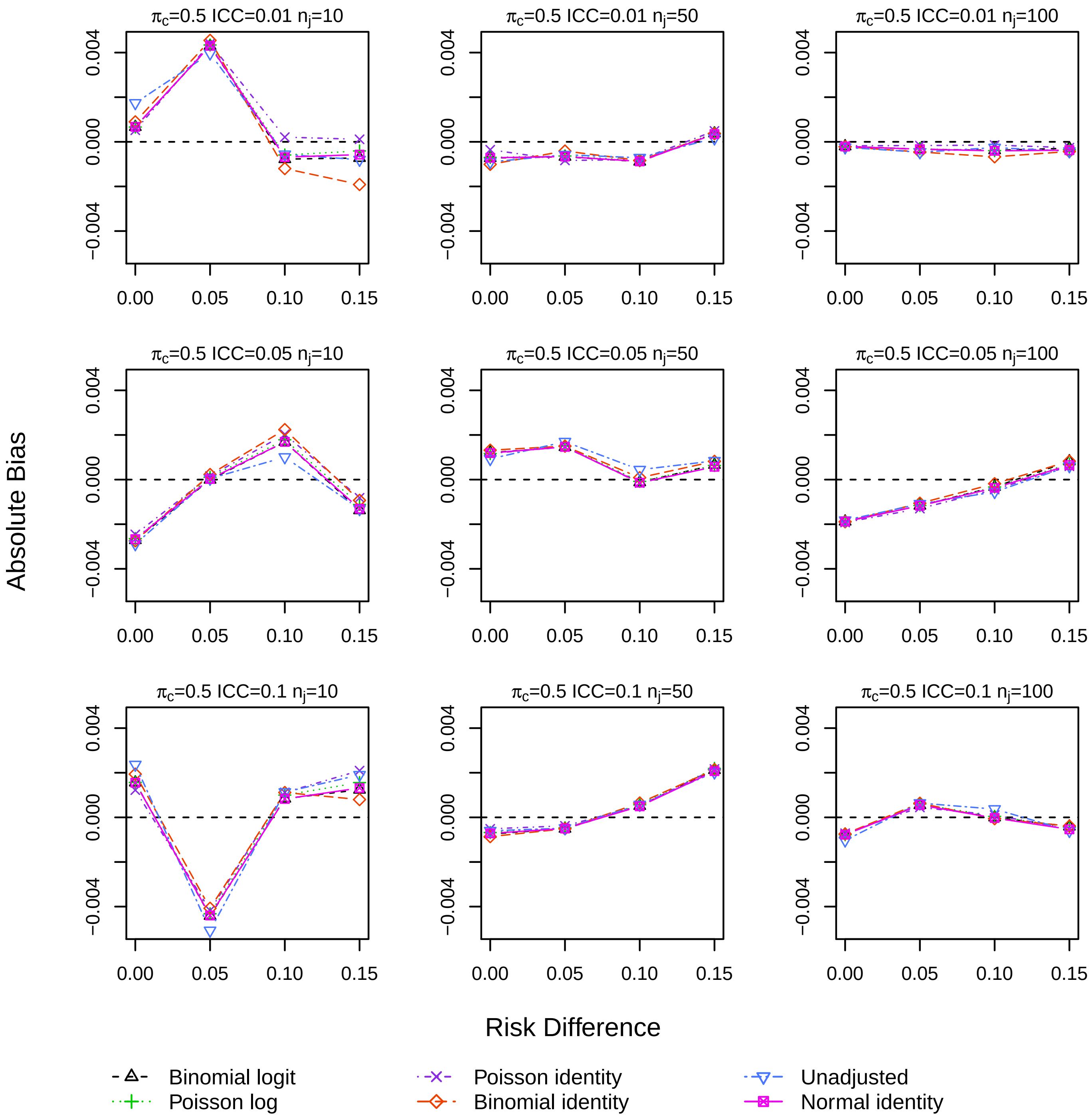


Figure S.9. Absolute bias for scenarios with true identity link function including a baseline covariate for $\pi_c = 0.50$.

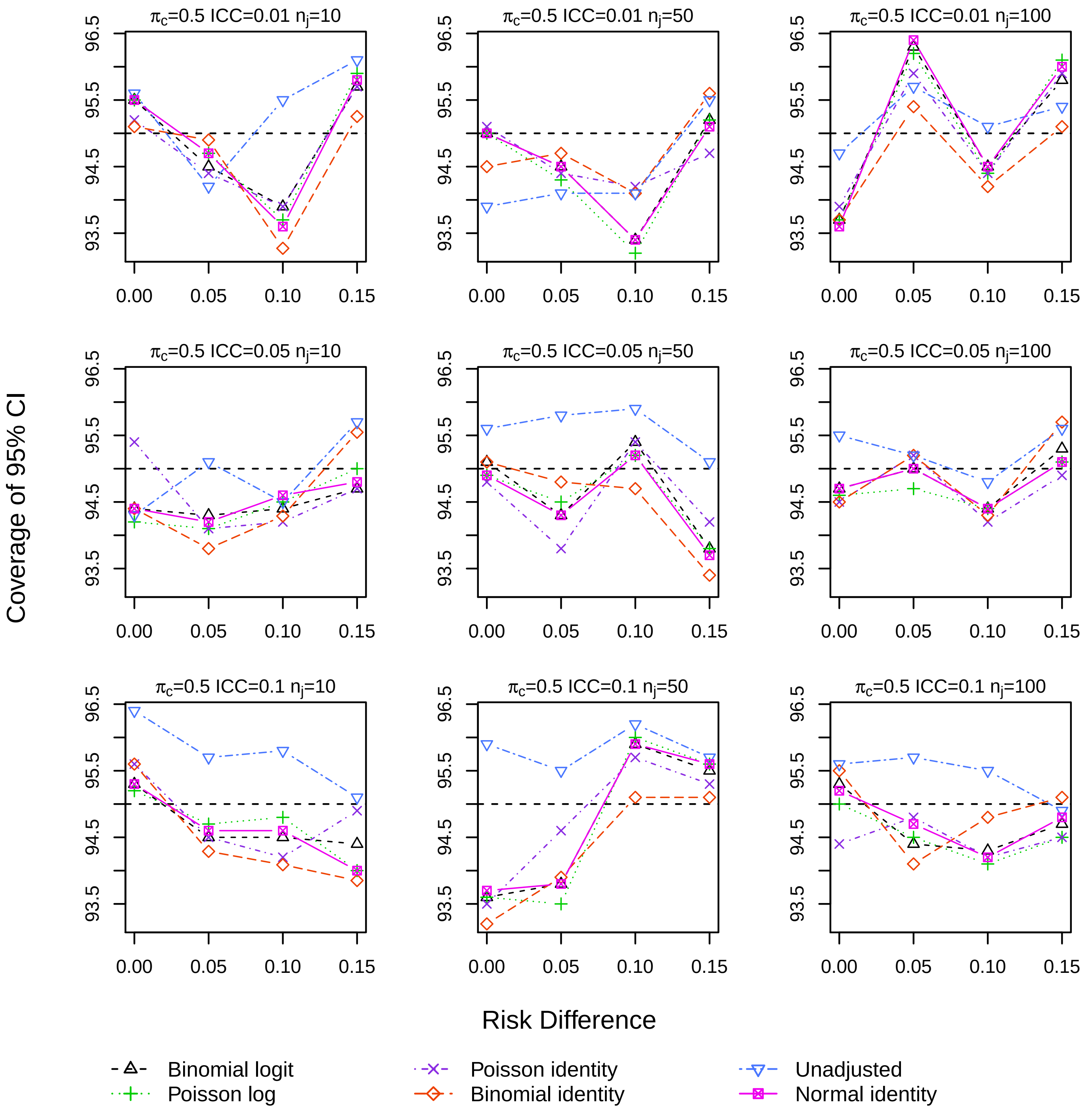


Figure S.10. Coverage of 95% CIs for scenarios with true identity link function including a baseline covariate for $\pi_c = 0.50$.

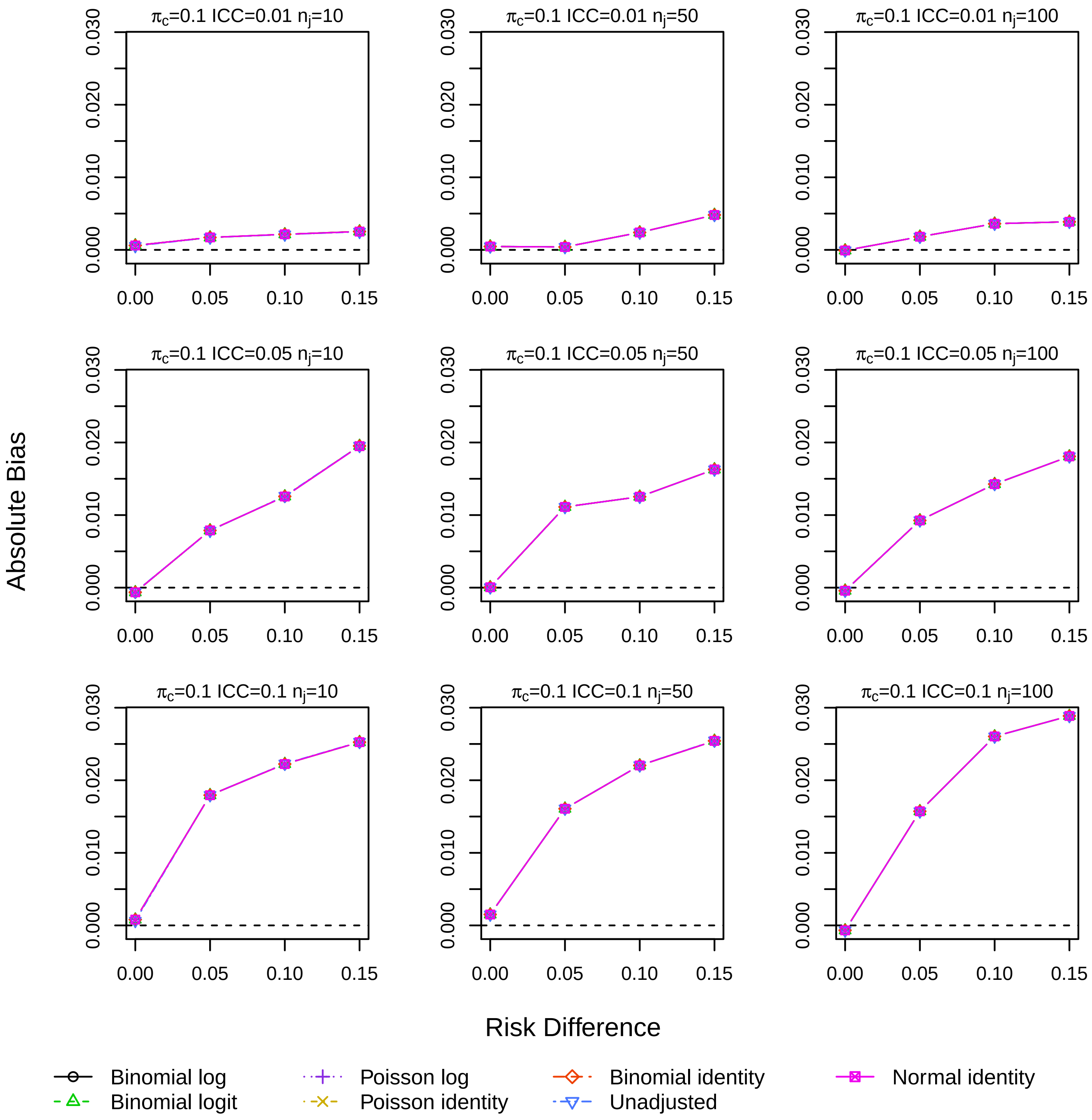


Figure S.11. Absolute bias for scenarios with true log link function without a baseline covariate for $\pi_c = 0.10$.

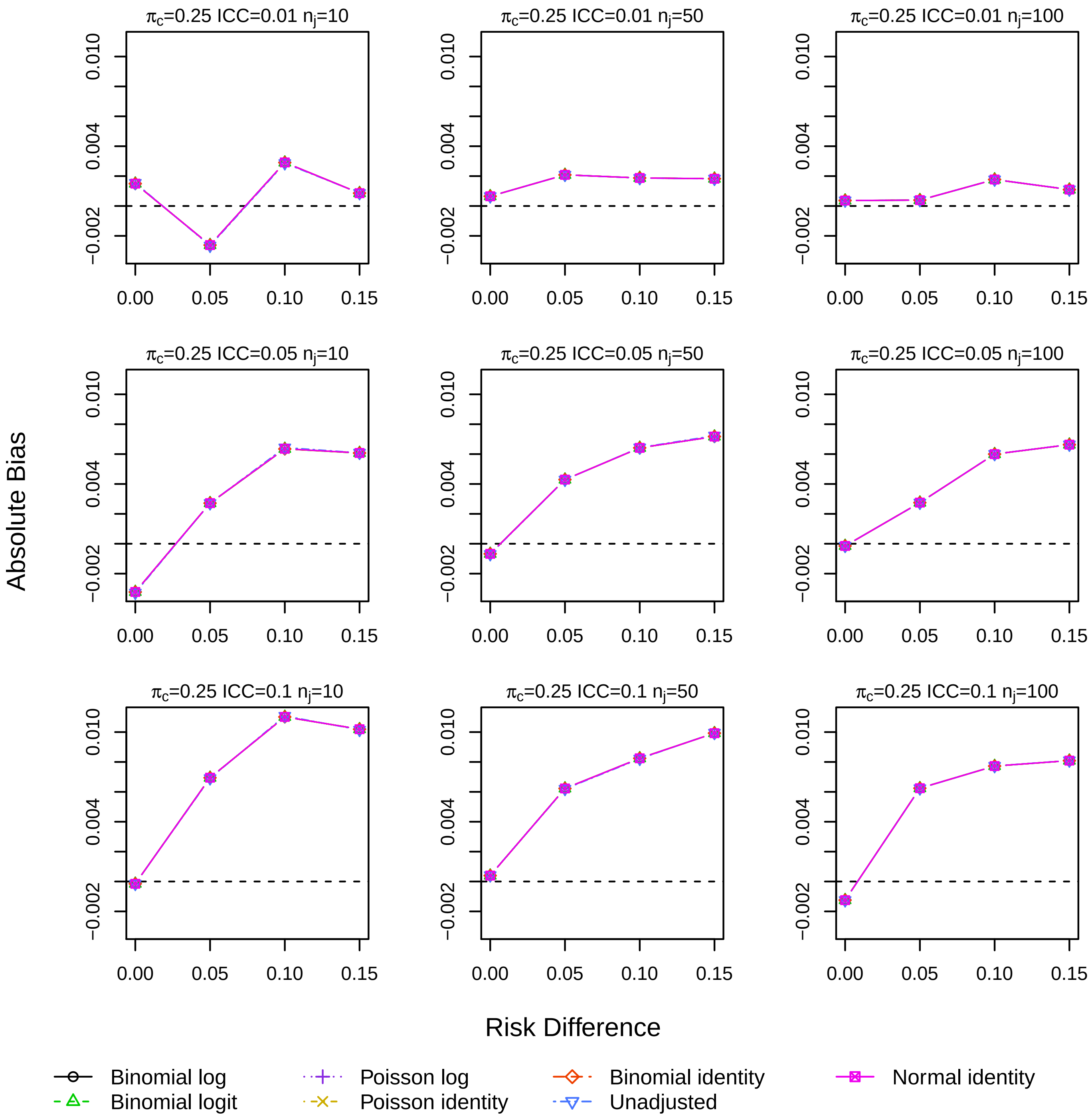


Figure S.12. Absolute bias for scenarios with true log link function without a baseline covariate for $\pi_c = 0.25$.

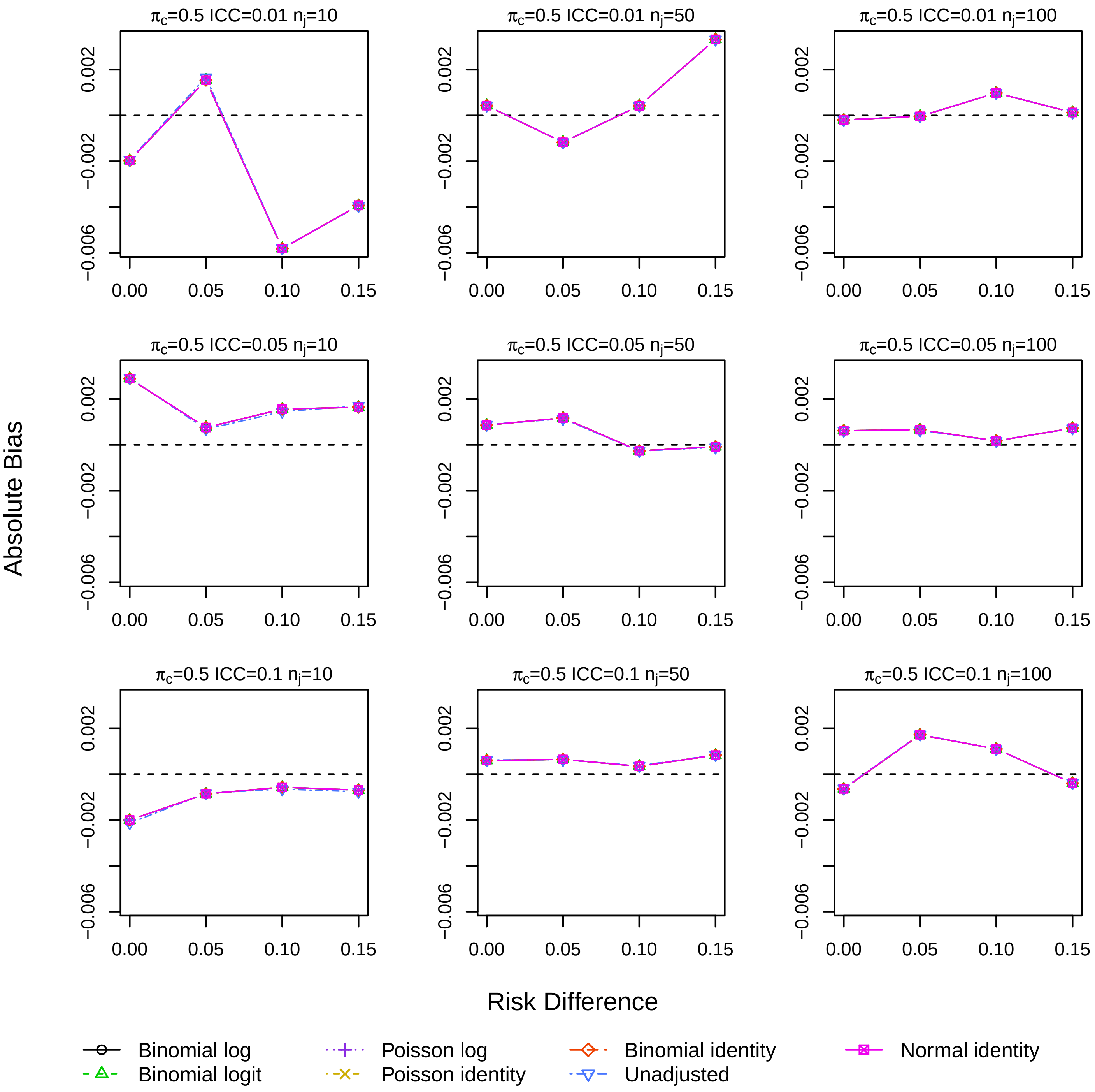


Figure S.13. Absolute bias for scenarios with true log link function without a baseline covariate for $\pi_c = 0.50$.

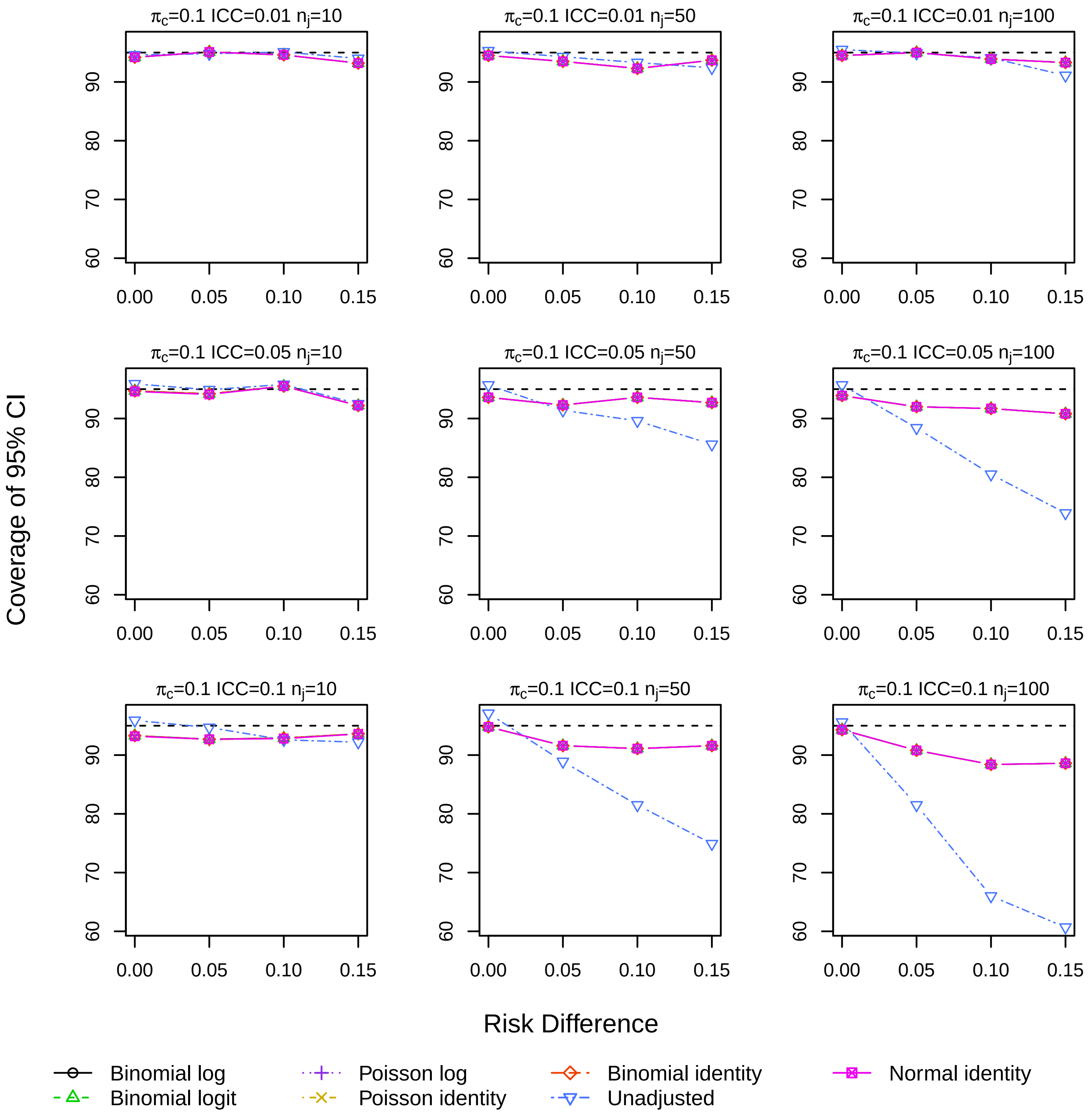


Figure S.14. Coverage of 95% CIs for scenarios with true log link function without a baseline covariate for $\pi_c = 0.10$.

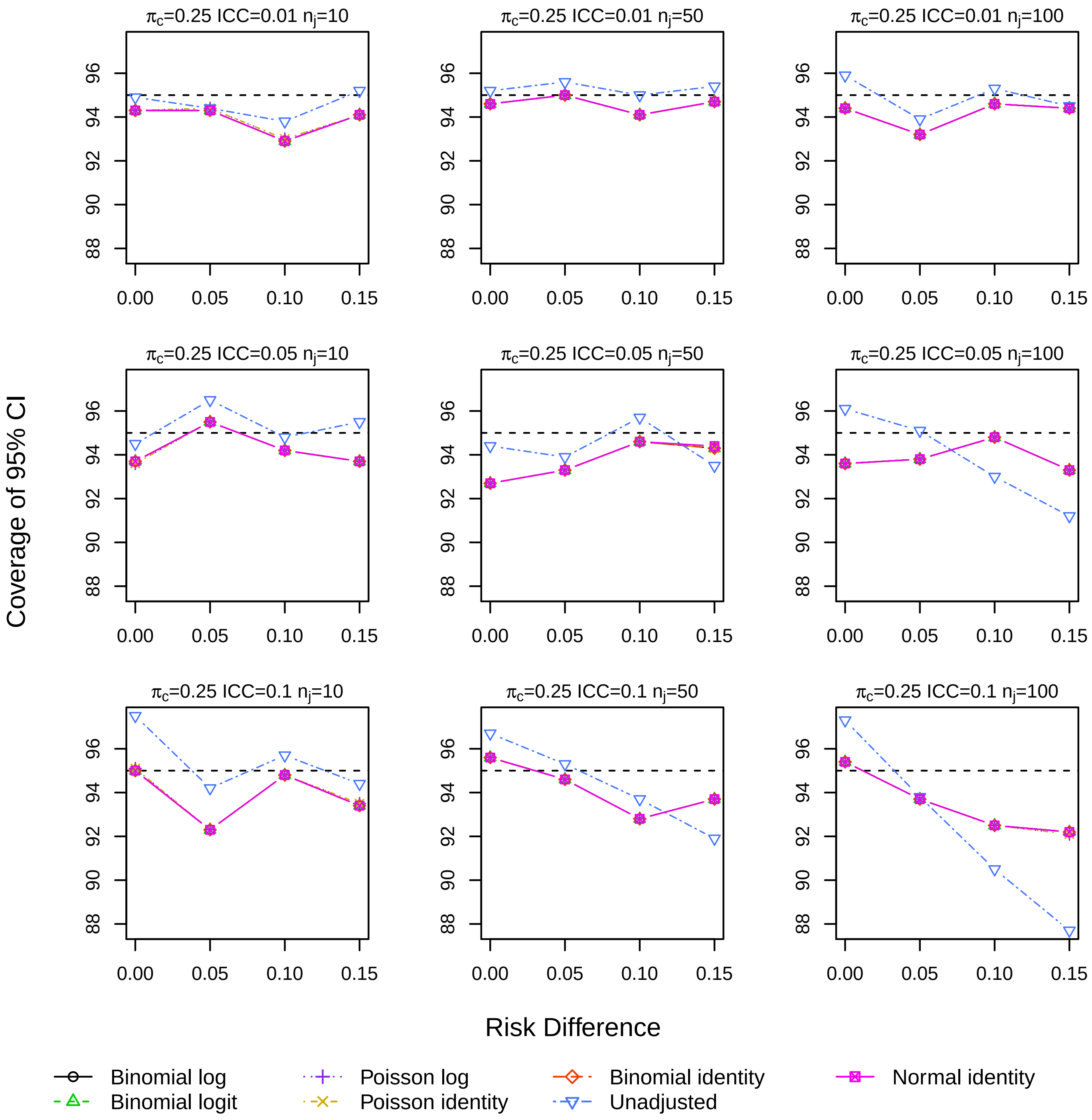


Figure S.15. Coverage of 95% CIs for scenarios with true log link function without a baseline covariate for $\pi_c = 0.25$.

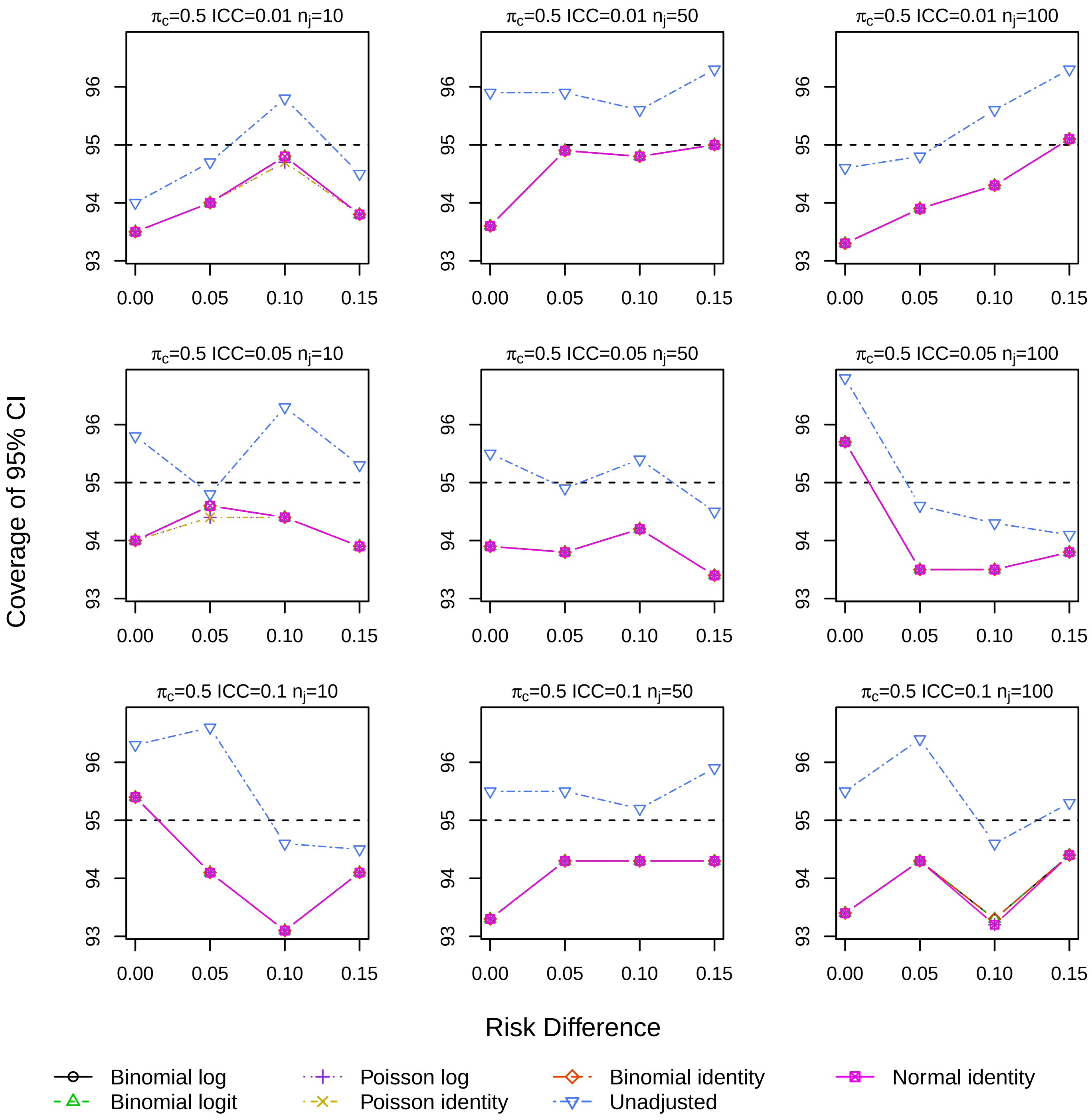


Figure S.16. Coverage of 95% CIs for scenarios with true log link function without a baseline covariate for $\pi_c = 0.50$.

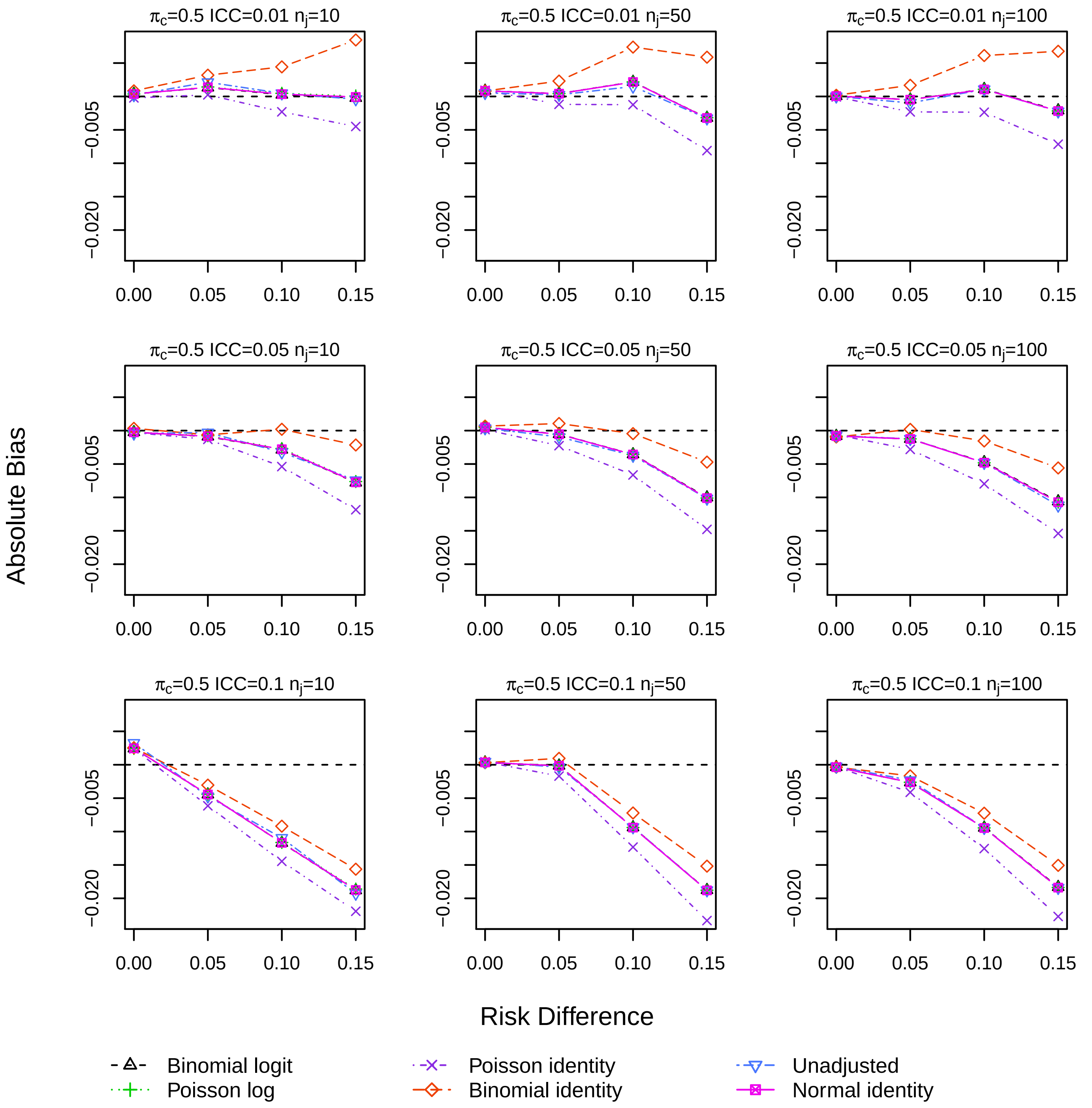


Figure S.17. Absolute bias for scenarios with true log link function including a baseline covariate for $\pi_c = 0.50$.

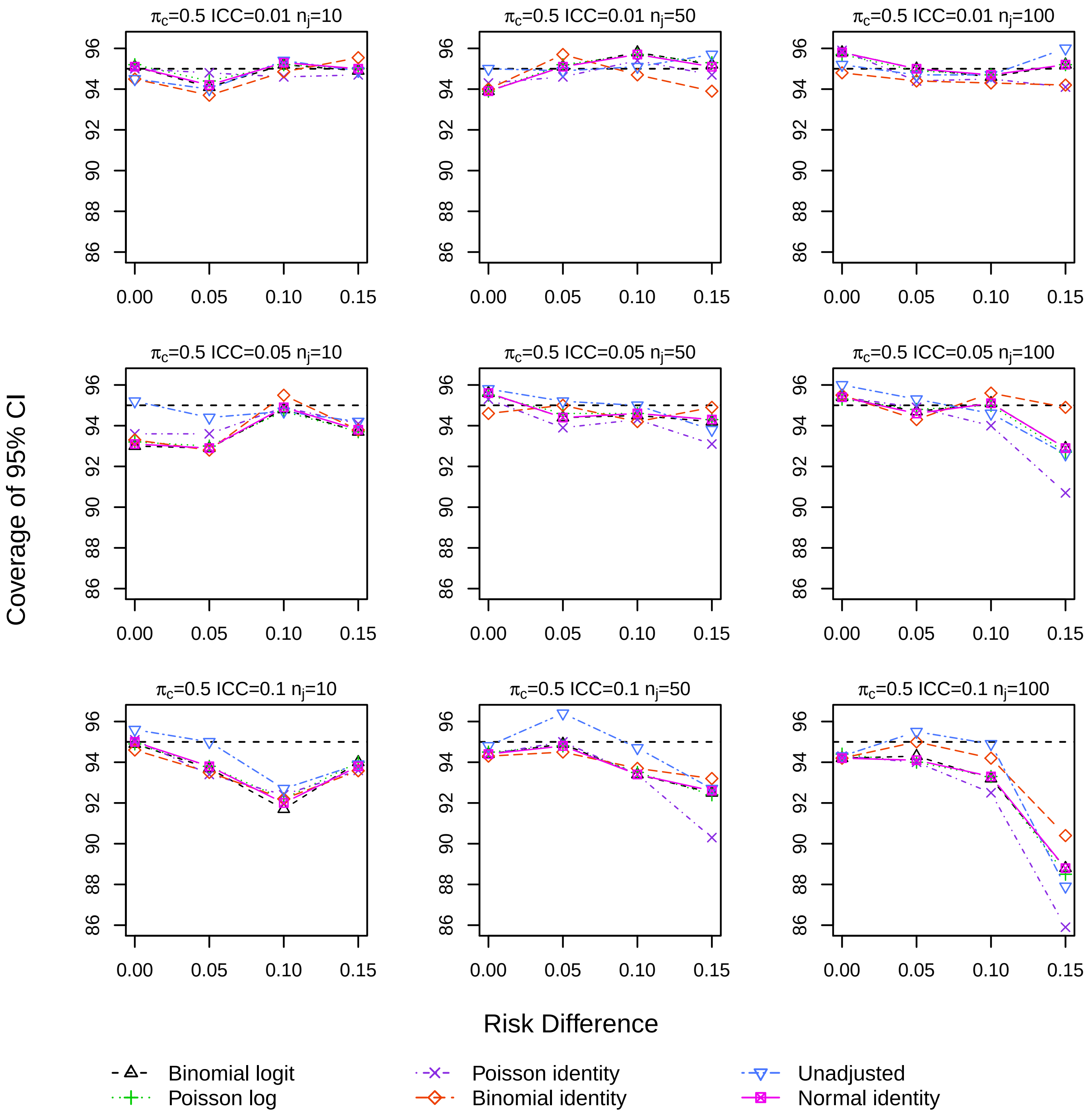


Figure S.18. Coverage of 95% CIs for scenarios with true log link function including a baseline covariate for $\pi_c = 0.50$.

# 2 Sample R code for calculating absolute risk difference adjusting for a baseline covariate

To use the functions below, the library `geepack` must be loaded first. We assume that the data consists of the binary outcome variable ‘y’, treatment group variable ‘treatment’, baseline predictor ‘z’, and cluster variable ‘center.’ Input for the functions is the dataset (sorted by center id).

## R functions to fit GEE models presented in the paper

```
library(geepack)

#BINOMIAL - IDENTITY MODEL
binIden<-function(data){
  fit1<-geese(y~treatment+z,id=center,family=binomial(link="identity"),
              corstr="exchangeable",data=data)

  ncenter<-length(unique(data$center))   #number of centers
  scf<-ncenter/(ncenter-2-1)         #small sample correction factor

  se.rd<-sqrt(diag(fit1$vbeta)[2]*scf)

  lower<-fit1$beta[2]-1.96*se.rd
  upper<-fit1$beta[2]+1.96*se.rd

  res<-c(fit1$beta[2],se.rd,lower,upper,fit1$alpha)
  names(res)<-c("rd","se.rd","lower","upper","ICC")
  return(res)
}

#----------------------------------------------------------------------
##POISSON - IDENTITY
poissonIden<-function(data){
  fit1<-geese(y~treatment+z,id=center,family=poisson(link="identity"),
              corstr="exchangeable",data=data)

  ncenter<-length(unique(data$center))   #number of centers
  scf<-ncenter/(ncenter-2-1)         #small sample correction factor

  se.rd<-sqrt(diag(fit1$vbeta)[2]*scf)

  lower<-fit1$beta[2]-1.96*se.rd
  upper<-fit1$beta[2]+1.96*se.rd
```

```
  res<-c(fit1$beta[2],se.rd,lower,upper,fit1$alpha)
  names(res)<-c("rd","se.rd","lower","upper","ICC")
  return(res)
}

#---------------------------------------------------------------------
## NORMAL - IDENTITY
normIden<-function(data){
  fit1<-geese(y~treatment+z,id=center,
              corstr="exchangeable",data=data)

  ncenter<-length(unique(data$center))   #number of centers
  scf<-ncenter/(ncenter-2-1)         #small sample correction factor

  se.rd<-sqrt(diag(fit1$vbeta)[2]*scf)

  lower<-fit1$beta[2]-1.96*se.rd
  upper<-fit1$beta[2]+1.96*se.rd

  res<-c(fit1$beta[2],se.rd,lower,upper,fit1$alpha)
  names(res)<-c("rd","se.rd","lower","upper","ICC")
  return(res)
}

#---------------------------------------------------------------------

## BINOMIAL  LOG MODEL
binlog<-function(data){
  fit1<-geese(y~treatment+z,id=center,family=binomial(link="log"),
              corstr="exchangeable",data=data)
  betas<-fit1$beta
  zvar<-data$z

  ncenter<-length(unique(data$center)) #number of centers
  scf<-ncenter/(ncenter-2-1)         #small sample correction factor

  pc<-mean(exp(betas[1]+betas[3]*zvar))
  pt<-mean(exp(betas[1]+betas[2]+betas[3]*zvar))
  rd<-pt-pc

  d1<-mean(exp(betas[1]+betas[2]+betas[3]*zvar) - exp(betas[1]+betas[3]*zvar))
  d2<-mean(exp(betas[1]+betas[2]+betas[3]*zvar))
  d3<-mean(exp(betas[1]+betas[2]+betas[3]*zvar)*zvar -
             exp(betas[1]+betas[3]*zvar)*zvar)
```

```
  dvec<-c(d1,d2,d3)

  var.pdiff<-dvec%*%fit1$vbeta%*%dvec
  se.rd<-sqrt(var.pdiff*scf)

  lower<-rd-1.96*se.rd
  upper<-rd+1.96*se.rd

  res<-c(rd,se.rd,lower,upper,fit1$alpha)
  names(res)<-c("rd","se.rd","lower","upper","ICC")
  return(res)
}

#-------------------------------------------------------------
### POISSON - LOG

poissonlog<-function(data){
  fit1<-geese(y~treatment+z,id=center,family=poisson(link="log"),
              corstr="exchangeable",data=data)
  betas<-fit1$beta
  zvar<-data$z

  ncenter<-length(unique(data$center)) #number of centers
  scf<-ncenter/(ncenter-2-1)         #small sample correction factor

  pc<-mean(exp(betas[1]+betas[3]*zvar))
  pt<-mean(exp(betas[1]+betas[2]+betas[3]*zvar))
  rd<-pt-pc

  d1<-mean(exp(betas[1]+betas[2]+betas[3]*zvar) - exp(betas[1]+betas[3]*zvar))
  d2<-mean(exp(betas[1]+betas[2]+betas[3]*zvar))
  d3<-mean(exp(betas[1]+betas[2]+betas[3]*zvar)*zvar -
           exp(betas[1]+betas[3]*zvar)*zvar)
  dvec<-c(d1,d2,d3)

  var.pdiff<-dvec%*%fit1$vbeta%*%dvec
  se.rd<-sqrt(var.pdiff*scf)
  lower<-rd-1.96*se.rd
  upper<-rd+1.96*se.rd

  res<-c(rd,se.rd,lower,upper,fit1$alpha)
  names(res)<-c("rd","se.rd","lower","upper","ICC")
  return(res)
}
```

```
#-----------------------------------------------------------------
##BINOMIAL - LOGIT
binlogit<-function(data){
  fit1<-geese(y~treatment+z,id=center,family=binomial(link="logit"),
              corstr="exchangeable",data=data)
  betas<-fit1$beta
  zvar<-data$z

  ncenter<-length(unique(data$center)) #number of centers
  scf<-ncenter/(ncenter-2-1)         #small sample correction factor

  pc<-mean(invlogit(betas[1]+betas[3]*zvar))
  pt<-mean(invlogit(betas[1]+betas[2]+betas[3]*zvar))
  rd<-pt-pc

  d1<-mean(exp(-(betas[1]+betas[2]+betas[3]*zvar))/
            (1+exp(-(betas[1] + betas[2] + betas[3]*zvar)))^2 -
            exp(-(betas[1] + betas[3] *zvar))/
            (1 + exp(-(betas[1] +betas[3]*zvar)))^2)
  d2<-mean(exp(-(betas[1]+betas[2]+betas[3]*zvar))/
            (1 +exp(-(betas[1] +betas[2]+betas[3]*zvar)))^2)
  d3<-mean(exp(-(betas[1]+betas[2]+betas[3]*zvar))* zvar/
            (1 +exp(-(betas[1] +betas[2]+betas[3]*zvar)))^2
          - exp(-(betas[1]+betas[3]*zvar))*zvar/
            (1+exp(-(betas[1]+betas[3]*zvar)))^2)
  dvec<-c(d1,d2,d3)

  var.pdiff<-dvec%*%fit1$vbeta%*%dvec
  se.rd<-sqrt(var.pdiff*scf)

  lower<-rd-1.96*se.rd
  upper<-rd+1.96*se.rd

  res<-c(rd,se.rd,lower,upper,fit1$alpha)
  names(res)<-c("rd","se.rd","lower","upper","ICC")
  return(res)
}
```
